# Phase-separated ParB enforces diverse DNA compaction modes and stabilizes the parS-centered partition complex

**DOI:** 10.1093/nar/gkae533

**Published:** 2024-06-22

**Authors:** Yilin Zhao, Lijuan Guo, Jiaojiao Hu, Zhiyun Ren, Yanan Li, Meng Hu, Xia Zhang, Lulu Bi, Dan Li, Hanhui Ma, Cong Liu, Bo Sun

**Affiliations:** School of Life Science and Technology, ShanghaiTech University, Shanghai 201210, China; School of Life Science and Technology, ShanghaiTech University, Shanghai 201210, China; Interdisciplinary Research Center on Biology and Chemistry, Shanghai Institute of Organic Chemistry, Chinese Academy of Sciences, Shanghai 201210, China; State Key Laboratory of Chemical Biology, Shanghai Institute of Organic Chemistry, Chinese Academy of Sciences, Shanghai 200032, China; School of Life Science and Technology, ShanghaiTech University, Shanghai 201210, China; CAS Center for Excellence in Molecular Cell Science, Shanghai Institute of Biochemistry and Cell Biology, Chinese Academy of Sciences, Shanghai 200031, China; University of Chinese Academy of Sciences, Beijing 100049, China; School of Life Science and Technology, ShanghaiTech University, Shanghai 201210, China; School of Life Science and Technology, ShanghaiTech University, Shanghai 201210, China; School of Life Science and Technology, ShanghaiTech University, Shanghai 201210, China; School of Life Science and Technology, ShanghaiTech University, Shanghai 201210, China; Bio-X Institutes, Key Laboratory for the Genetics of Developmental and Neuropsychiatric Disorders, Ministry of Education, Shanghai Jiao Tong University, Shanghai 200240, China; Zhangjiang Institute for Advanced Study, Shanghai Jiao Tong University, Shanghai 200240, China; School of Life Science and Technology, ShanghaiTech University, Shanghai 201210, China; Interdisciplinary Research Center on Biology and Chemistry, Shanghai Institute of Organic Chemistry, Chinese Academy of Sciences, Shanghai 201210, China; State Key Laboratory of Chemical Biology, Shanghai Institute of Organic Chemistry, Chinese Academy of Sciences, Shanghai 200032, China; School of Life Science and Technology, ShanghaiTech University, Shanghai 201210, China

## Abstract

The tripartite ParAB*S* system mediates chromosome segregation in the majority of bacterial species. Typically, DNA-bound ParB proteins around the *parS* sites condense the chromosomal DNA into a higher-order multimeric nucleoprotein complex for the ParA-driven partition. Despite extensive studies, the molecular mechanism underlying the dynamic assembly of the partition complex remains unclear. Herein, we demonstrate that Bacillus subtilis ParB (Spo0J), through the multimerization of its N-terminal domain, forms phase-separated condensates along a single DNA molecule, leading to the concurrent organization of DNA into a compact structure. Specifically, in addition to the co-condensation of ParB dimers with DNA, the engagement of well-established ParB condensates with DNA allows for the compression of adjacent DNA and the looping of distant DNA. Notably, the presence of CTP promotes the formation of condensates by a low amount of ParB at *parS* sites, triggering two-step DNA condensation. Remarkably, *parS*-centered ParB-DNA co-condensate constitutes a robust nucleoprotein architecture capable of withstanding disruptive forces of tens of piconewton. Overall, our findings unveil diverse modes of DNA compaction enabled by phase-separated ParB and offer new insights into the dynamic assembly and maintenance of the bacterial partition complex.

## Graphical Abstract

**Graphical Abstract:**
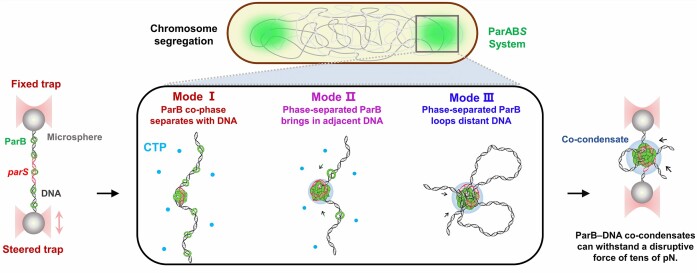

## Introduction

Chromosome segregation, a process that redistributes newly replicated genomic material to daughter cells, is crucial for maintaining genetic integrity and stability in all domains of life (1,2). In two-thirds of bacterial species, the accurate segregation of chromosomes and low-copy-number plasmids relies on the tripartite ParAB*S* system, consisting of the ATPase protein ParA, the DNA-binding protein ParB, and the centromeric-like, *cis*-acting *parS* DNA sequence (3,4). The short palindromic *parS* sites are often located near the origins of replication on the chromosome (5). ParB proteins first recognize and specifically bind to *parS* sites in coordination with DNA replication, followed by laterally spreading to adjacent non-specific (ns) DNA (6–8). The properly distributed ParB proteins condense the chromosomal DNA into a poorly organized multimeric nucleoprotein complex, forming the partition entity (8–12). This partition complex is a functional assembly that can dynamically interact with the P-loop ATPase ParA (13–15). The stimulated ATP activity of ParA creates a gradient of nucleoid-associated ParA–ATP dimers, providing the pulling force that drives the movement of the kinetochore-like partition complex toward the cell poles (16,17). During segregation, the formation of the ParB–DNA partition complex is key to the faithful redistribution of chromosomes. Substantial efforts have been devoted to understanding the ParB protein and its dynamic interactions with DNA.

Members of the ParB superfamily share a conserved functional architecture: a C-terminal domain (CTD) for dimerization, a central helix–turn–helix (HTH) domain for DNA binding (CDBD), and an N-terminal domain (NTD) for protein–protein interactions (9,18). In addition to multimerization and ParA interactions, the NTD of ParB has also been acknowledged to contain a CTP-binding pocket that allows for CTP binding and hydrolysis (19,20). Once bound by CTP, dimeric ParB forms a clamp-like structure that encircles DNA in the center of the clamp (21–23). This structural change weakens the association of ParB dimers with *parS* sites and licenses their lateral spreading to access neighboring DNA (21,22). While maintaining association with DNA, CTP hydrolysis triggers the clamp opening of ParB dimers at the N-terminus and permits DNA bridging via a ParB–ParB interaction (21,24). It is also noteworthy that CTP is one of the determinants for the partition complex assembly. Its presence can significantly decrease the ParB concentration required for DNA condensation *in vitro* (22,24–26).

Beginning with *parS* binding and spreading, how DNA-bound ParB dimers further generate a higher-order network of the ParB–DNA complex has been implicated in a few proposed models, including ‘nucleation and caging’ and phase separation (27–29). Although the precise mechanism is still under debate, evidence is accumulating that the formation of the loosely organized ParB–DNA network necessarily requires the coalescence of multiple ParB dimers into co-condensates with DNA, wherein the multimerization interfaces between the NTDs of the ParB proteins are indispensable (11,27,29). First, the ChIP-seq analysis supports the aggregation of ParB around *parS* sites in various bacterial species (6). Second, our recent single-molecule study has proved that ParB dimers can stochastically multimerize along nsDNA (30). The ParB multimer prefers to remain immobile on *parS* sites and can bridge and translocate other DNA molecules (30). Importantly, the condensation of a DNA molecule containing a single *parS* site has recently been realized under a low ParB concentration (25). This process also necessitates the multimerization of ParB dimers. Third, super-resolution microscopy has unveiled an *in vivo* nucleation process characterized by forming nanometer-sized condensates involving hundreds of *Escherichia coli* F plasmid ParB molecules and *parS*-containing DNA (28). In line with these findings, *C. glutamicum* ParB possesses an intrinsic ability to co-phase separate with DNA *in vitro*, which is also modulated by CTP (29). Fourth, theoretical frameworks also established the condensate formation led by *parS* nucleated phase transition (27,29,31,32). Inspired by these findings, many questions regarding the assembly and maintenance of the partition complex have been put forth. For instance, whether and if yes, how does the phase separation of ParB drive the DNA condensation? What are the respective roles of ParB dimer and multimer in forming the partition complex? How is the *parS*-centered structure formed and maintained? How do *parS* and CTP promote DNA condensation by a low amount of ParB proteins?

Taking *Bacillus subtilis* (Bs) ParB (known as Spo0J, hereafter referred to as ParB) as a model, we aimed to address the abovementioned questions in this study. We demonstrated that ParB has the inherent ability to undergo phase separation and to form co-condensates with DNA. By combining optical tweezers with confocal microscopy, we further examined the role of phase-separated ParB in the dynamic assembly of the partition complex at the single-molecule level. Interestingly, ParB phase separation drives DNA compaction and condensation in several ways. Moreover, the presence of CTP promotes the coacervation of ParB molecules at *parS* sites, which leads to a *parS*-centered, two-step DNA condensation process. Notably, the ParB–DNA co-condensates at *parS* motifs can tolerate a disruptive force of up to 50 pN and thus possibly contribute to stabilizing the global nucleoprotein architecture. These findings deepen our understanding of bacterial partition complex assembly, and a model of phase-separated ParB organizing DNA into a *parS*-centered, higher-order structure has been proposed.

## Materials and methods

### Expression and purification of ParB proteins

The procedures for ParB expression and purification are as follows. The *parB* gene was initially amplified and inserted into a pET-based expression vector featuring an N-terminal His_6_-tag. *Escherichia coli* BL21 (DE3) (TransGen) cells were utilized for protein expression. The cells were cultivated in an LB medium containing kanamycin at 37°C until the optical density at 600 nm reached 0.6. Protein expression was induced by adding 1 mM isopropyl-beta-d-thiogalactopyranoside and continued at 16°C for 16 hours. The harvested cells were lysed in a solution comprising 1 mM EDTA, 500 mM NaCl, 50 mM Tris–HCl pH 7.5, and 1% protease inhibitor. The lysate was then filtered through a 0.22-μm Millipore Express® PES Membrane Filter Unit and disrupted using a high-pressure cell crusher at approximately 800 bar. The resulting lysate was centrifuged at 16639 g for 30 minutes to obtain a clarified supernatant, which was subsequently batch-bound to Ni-NTA Agarose (TransGen). The column was washed with a series of lysis buffers containing varying imidazole concentrations (5, 20, 50 and 200 mM), with the majority of ParB proteins being eluted at 200 mM imidazole. The eluate was then concentrated to a specific volume at 4°C using 30-kDa filtration at 4500 g. Further purification of ParB involved passage through a 5-mL HiTrap Heparin HP column (GE Healthcare) and gel filtration chromatography using a Superdex increase 200 10/30 column (GE Healthcare) in ParB storage buffer (50 mM Tris–HCl pH 7.5, 300 mM NaCl, and 10% (v/v) glycerol). The purified protein was stored at –80°C before use. The P93S variant of BsParB, an analogue to P107S from *Pseudomonas aeruginosa* that is defective in multimerization (33), was generated by site-directed mutagenesis in the protein sequence. P93S, ParB-eGFP, P93S-eGFP and ParB-ΔNTD-eGFP were expressed and purified similarly.

### *In vitro* phase separation assay

In the phase separation assays, samples of ParB-eGFP and P93S-eGFP were prepared in a buffer containing 50 mM Tris–HCl pH 7.5 and varying NaCl concentrations (100, 200 and 300 mM). For co-condensation studies, these proteins were individually mixed with 1 μM Cy5-labeled *parS* DNA or nsDNA. Following the initiation of phase separation, the mixtures were placed onto glass-bottom dishes (Model 80100, NEST). For the 1,6-hexanediol treatment, 1,6-hexanediol was added to the phase-condensed protein to reach a final 3% or 6% (v/v) solution. The phase-separated proteins were typically incubated at room temperature for 5 minutes before imaging unless otherwise stated. The samples were allowed to rest on the slide for 5 min for the droplets to settle at the bottom layer. This bottom layer was exclusively focused for imaging to ensure consistency. Phase separation was visualized using a Leica TCS SP8 microscope with a 100× oil immersion objective, acquiring confocal images at room temperature. The area of ParB droplets under each condition was quantified using ImageJ software. The image was first converted into an 8-bit image, followed by the threshold adjustment to 40 for a comprehensive selection of the droplets. The area was then measured using the ‘analyze particles’ function. Data analysis was conducted through a Two-sample t-test, with statistical significance set at *P* < 0.05 for all experiments.

### Fluorescence recovery after photobleaching (FRAP) assay

The FRAP assay was executed using the FRAP module on a Leica TCS SP8 confocal microscope with a 100× oil immersion objective. This procedure involved selectively bleaching fluorescently labeled assemblies with a laser beam, targeting a specific circular region of interest. Following photobleaching, imaging was performed continuously, capturing one frame every 2.58 seconds. The fluorescence intensity in the bleached region (*I*_t_^m^) and the intensity (*I*_t_^c^) in a nearby unbleached assembly serving as a control were measured. For quantitative analysis, the fluorescence intensity at the bleached site at each time point (t) was normalized against the control. The fluorescence recovery was calculated using the formula: *I*_t_= (*I*_t_^m^/*I*_0_^m^)/(*I*_t_^c^/*I*_0_^c^). All captured images were subsequently analyzed using the Leica Application Suite X software.

### Saturation concentration determination assay

The saturation concentration is defined as the critical protein concentration for phase separation, below which all proteins remained in the light phase (no aggregation). It is noteworthy that the concentration of the light-phased protein was maintained at the saturation concentration upon the initiation of condensation. A centrifugation method was utilized to determine the saturation concentration required for ParB condensation. Phase-separated ParB was first prepared in tubes at various concentrations in the specified buffer. Following a 10-min incubation, the ParB condensates were collected by centrifugation at 10 000 rpm for 10 min at 4°C. The concentration in the resulting clarified supernatant, the light phase, was then measured using a NanoDrop 2000C spectrophotometer (Thermo Scientific).

### Preparation of DNA templates

Oligonucleotides for the construction of the DNA segments used in the phase separation assays were purchased from Sangon Biotech (Supplementary Table S1). The Cy5-*parS* DNA and Cy5-nsDNA were produced by mixing Cy5-labeled DNA with complementary DNA at a molar ratio of 1:2 in the annealing buffer containing 50 mM Tris–HCl pH 7.5 and 100 mM NaCl. The mixtures were incubated at 95°C for 3 min and then slowly cooled to room temperature within 3 hours.

The λ phage DNA template was constructed as described elsewhere (34). Briefly, biotinylated λ DNA was built through 3′-end labeling by filling in 5′-overhangs with an exo-Klenow fragment. The reaction was conducted by incubating 10 nM λ DNA (Thermo Scientific), 250 μM dGTP/dATP/dTTP, 10 μM biotin-14-dCTP (Thermo Scientific) and 5 U Klenow exo^–^ (Thermo Scientific) in reaction buffer (50 mM NaCl, 10 mM Tris–HCl pH 7.9, 10 mM MgCl_2_ and 1 mM DTT) at 37°C for 1 h, followed by heat inactivation for 10 min at 75°C. The mixture was purified by Column DNA Purification Kit.

The DNA template containing 8× *parS* motifs was the ligation product of two DNA segments, L1 and L2. The 16x *parS* template was similarly prepared using L1 and L3 (Supplementary Figure S1). First, a 544-bp DNA segment containing 8x *parS* was PCR-amplified from the modified plasmid pDONOR4.1. This DNA segment was then inserted into a 13.4-kb lentiCRISPRi plasmid to generate a 13.8-kbp plasmid containing 8x *parS*. The 13.8-kb L1 and L3 DNA segments were PCR-amplified from the 13.8-kb plasmid containing 8× *parS*. The 13.3-kb L2 was PCR-amplified from λ DNA. The resulting DNA fragments were digested with XhoI (NEB) to create an overhang for ligation. The partial match of the two oligonucleotide sequences allows for two overhangs at each end for ligation by T4 DNA ligase (NEB). The 27.6-kb DNA template containing no *parS* motifs is PCR-amplified from λ DNA. All primer and oligonucleotide sequences used in this work are listed in Supplementary Table S1.

### Optical-tweezer-based single-molecule assays

A combination of a dual-optical tweezers setup, confocal microscopy, and microfluidics (LUMICKS, C-trap) was employed for the single-molecule measurements (35,36). Briefly, a 1064-nm fiber laser and a water-immersion objective were utilized to create two orthogonally polarized optical traps. The separation between the traps was controlled using a piezo mirror for beam-steering one trap. Force measurements were conducted by back-focal plane interferometry of the condenser top lens. The trap stiffness was typically set around 0.6 pN/nm and calibrated by measuring the effect of the optical trap on the Brownian motion of the trapped microsphere (37). A computer-controlled stage facilitated the rapid movement of the optical traps within a multiple-channel flow cell (34). This microfluidic system enabled the swift *in situ* construction and characterization of DNA dumbbell constructs and allowed for the efficient transfer of tethered DNA between different flow channels. DNA molecules were captured between two streptavidin-coated polystyrene beads (4.34 μm in diameter, Spherotech) using the multichannel laminar flow cell and were tensioned by increasing the length between the optical traps. A single DNA molecule was confirmed by its inherent mechanical force-extension relationship (38). After the confirmation, the DNA tether was transported to the protein or buffer channel, as described for each assay. During data acquisition, a constant (∼0.10 ± 0.08 pN) or increased force was applied to the DNA tether via a high-frequency feedback system on the steered trap. All experiments were conducted in 50 mM Tris–HCl pH 7.5, 150 mM NaCl, 1 mg/ml BSA, 1% Tween-20 and 4 mM MgCl_2_. DNA was stretched at 0.1 μm/s in the DNA relaxation and stretching assays. DNA force and extension data were recorded at a rate of 100 Hz.

Confocal scans were performed using 488 nm and 532 nm lasers for imaging ParB-eGFP and SYTOX-Orange stained DNA, respectively. Kymographs were generated via a confocal scan through the center of the two beads with a pixel size of 75 nm for 0.1 ms.

### Single-molecule data analysis

The single-molecule data were analyzed using custom software provided by LUMICKS. The ImageJ software was utilized to quantify the fluorescence intensity of the ParB multimer and DNA foci. The region of interest (ROI), approximately 6 × 6 pixels, was selected to encompass the ParB multimer or DNA foci. The fluorescence intensity within the ROI was summed up. The size of the ParB multimer was quantified using the full width at half maximum (FWHM) method (39). DNA-bound ParB multimers were selected, and an intensity profile was obtained along a line crossing the ParB multimer. The FWHM value was then calculated based on this intensity profile. The data were analyzed using a Two-sample t-test. A significance level of *P* < 0.05 was considered statistically significant for all experiments. The DNA extension was normalized by the length of the naked DNA.

## Results

### ParB undergoes phase separation via its NTD and forms co-condensates with DNA

**Figure 1.**
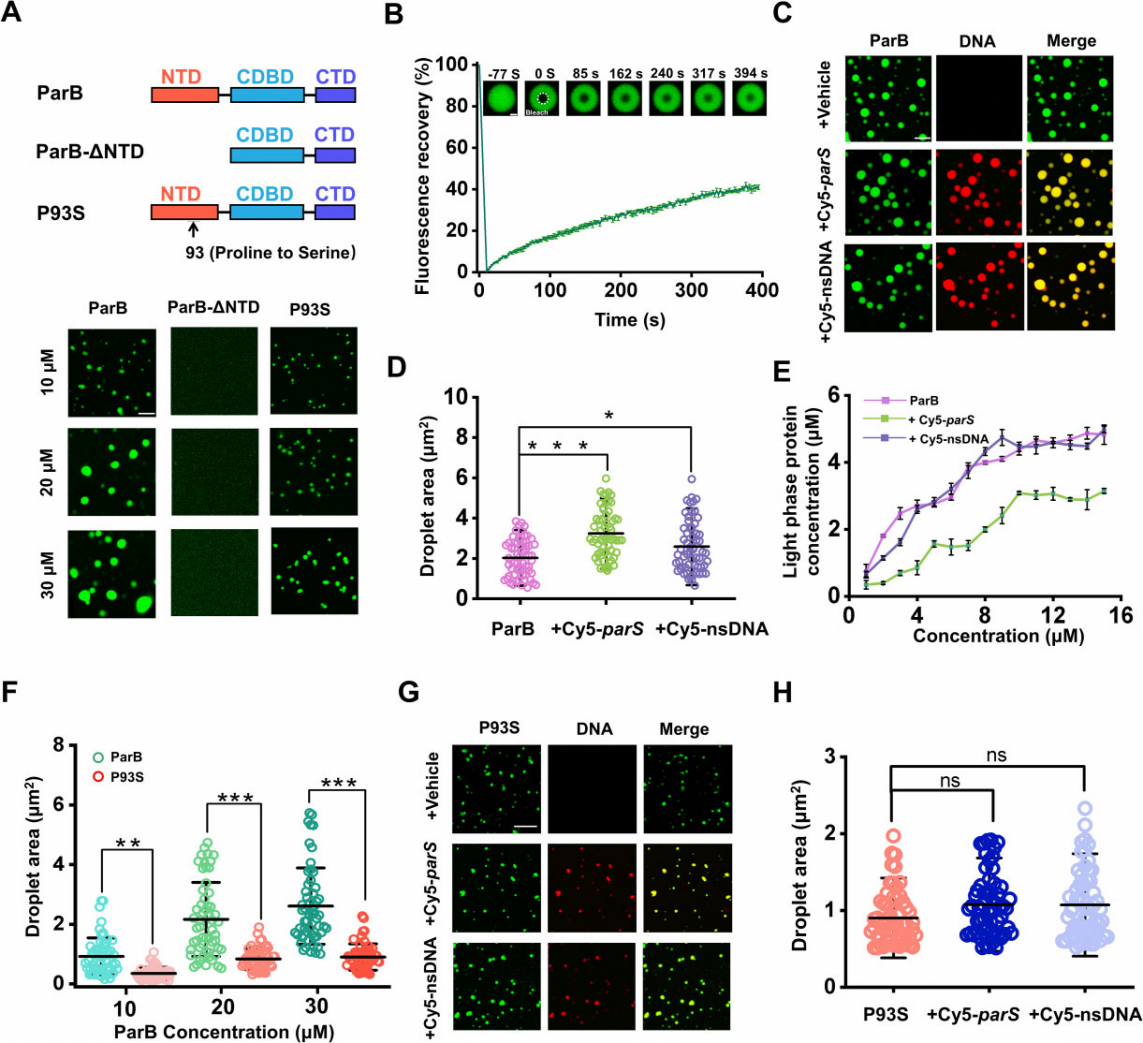
*In vitro* characterization of ParB condensation and its modulation by DNA. (**A**) (Top) Schematic representation of ParB, ParB-ΔNTD and P93S proteins. All ParB proteins are fused with eGFP for fluorescence visualization. (Bottom) Droplet formation by ParB, ParB-ΔNTD, and P93S across different protein concentrations. Scale bar, 5 μm. (**B**) (Top) Series of FRAP images illustrating ParB droplets formed under 40 μM with the bleached region indicated by a white dashed circle. Scale bar, 1 μm. (Bottom) Fluorescence recovery curves for ParB, with values shown as mean ± SD from six replicates. (**C**) Side-by-side images displaying co-condensation of 20 μM ParB-eGFP with and without 1 μM Cy5-parS or Cy5-nsDNA. The label ‘+vehicle’ signifies that the corresponding samples were treated with the DNA buffer containing 10 mM Tris–HCl pH 8.0 and 0.1 mM EDTA, which serves as the control solvent. Scale bar, 5 μm. (**D**) Statistical analysis of ParB droplet sizes under the conditions described in (C). Data are presented as mean ± SD, with *n* = 60 condensates analyzed across three independent experiments. *P < 0.05, ***P < 0.001. (**E**) Assessment of saturation concentrations of ParB in the absence and presence of Cy5-*parS* or Cy5-nsDNA, with error bars representing SD from three experiments. (**F**) Quantitative comparison of droplet sizes for ParB and P93S in the absence of DNA. Data are presented as mean ± SD, with n = 60 condensates analyzed across three independent experiments. **P < 0.01, ***P < 0.001. (**G**) Co-condensation images of 20 μM P93S with 1 μM Cy5-*parS* or Cy5-nsDNA. Scale bar, 5 μm. (**H**) Quantitative analysis of P93S droplet sizes under the experimental setups in (**G**), with data shown as mean ± SD for 60 replicates. ns, no significance.

Since both *in vitro* and *in vivo* evidence supports a model of ParB organizing partition complex assembly via phase separation (28,29), we first aimed to address whether the ability to undergo phase separation is conserved for *Bacillus subtilis* ParB. As shown in Figure 1A, ParB fused with eGFP (ParB-eGFP) forms spherical condensates at various concentrations with the sizes of these droplets notably increasing with protein concentration. To investigate the dynamics of these ParB condensates, we conducted fluorescence recovery after photobleaching (FRAP) experiments. These experiments revealed that the fluorescence intensity of ParB-eGFP clusters returned to around 40% within 400 seconds after photobleaching, suggesting these aggregates possess liquid-like characteristics (Figure 1B). Additionally, varying the incubation temperature (4–20°C) and time (5–30 minutes) had a negligible effect on the properties of the ParB droplets (Supplementary Figure S2A–C). However, the condensates were irregular at 40°C, showing obvious coalescence without any dynamic changes (Supplementary Figure S2A-C). To determine the driving forces responsible for ParB condensation, we increased the NaCl concentration to perturb electrostatic interactions and incorporated 1,6-hexanediol to attenuate hydrophobic interactions. The results indicated that electrostatic interactions predominantly facilitate ParB phase separation, with hydrophobic interactions playing a supplementary role (Supplementary Figure S2D, E).

As evidenced by previous studies (27,40–44), the *parS* motifs within DNA serve as the nucleation center of the partition complex. This characteristic led us to examine how *parS* DNA affects ParB condensation. Notably, we observed that ParB-eGFP undergoes co-phase separation with a 52-bp Cy5-labeled *parS*-containing DNA segment (Cy5-*parS*) (Supplementary Table S1), resulting in a noticeable increase in the size of the droplet (Figure 1C, D). We also examined the impact of a Cy5-labeled nsDNA fragment of the same length, lacking *parS* motifs (Cy5-nsDNA) (Supplementary Table S1). In contrast, Cy5-nsDNA exhibited a minimal effect on promoting ParB condensation, even though it was co-condensed with ParB. Notably, Cy5-*parS* reduced the saturation concentration of ParB for condensation from around 5 to 3 μM, an effect not seen with Cy5-nsDNA (Figure 1E). This discrepancy suggests that the specific binding of ParB to *parS* plays a crucial role in organizing chromosomal DNA.

The NTD of ParB is known to mediate dimer–dimer interactions and is crucial for protein multimerization (45). Therefore, we hypothesized that the NTD is essential for ParB condensation. To test this, we generated a ParB mutant lacking the NTD, termed ParB-ΔNTD (Figure 1A). We found that eGFP-fused ParB-ΔNTD (ParB-ΔNTD-eGFP) failed to form droplets under any tested conditions (up to 30 μM final concentration), even in the presence of Cy5-*parS* or Cy5-nsDNA (Figure 1A and Supplementary Figure S3). This finding indicates that removing the NTD impairs the abilities of ParB to condense and to co-phase separate with DNA.

We further set out to identify essential residues in the NTD of ParB that are involved in phase separation. A previous study on ParB from *Pseudomonas aeruginosa* demonstrated the impaired multimerization of ParB when key amino acids in the NTD were mutated, such as G71S, A97T, and P107S (33). Accordingly, we further explored the condensation properties of the P93S variant of BsParB, an analogue to P107S from *Pseudomonas aeruginosa*. Intriguingly, while P93S-eGFP could still form droplets, its phase separation was significantly attenuated, as evidenced by the smaller size of these droplets compared to those formed by the ParB-eGFP at equivalent concentrations (Figure 1A and F). Both Cy5-*parS* and Cy5-nsDNA were still able to co-phase separate with P93S-eGFP. However, the presence of the DNA did not significantly alter the phase separation of P93S-eGFP, with droplet sizes remaining similar to those in the absence of DNA (Figure 1G, H).

In summary, ParB, particularly through its NTD, can undergo co-phase separation with DNA, and *parS* DNA specifically enhances ParB’s phase-separation capacity.

### Two modes of DNA compaction enabled by phase-separated ParB multimer

Co-phase separation of ParB with DNA raised a possibility of DNA condensation driven by phase-separated ParB. Next, we employed a single-molecule approach to examine the dynamic formation of ParB–DNA co-condensates. This method combines optical tweezers with confocal microscopy to record a single suspended DNA molecule's length and force in real time and simultaneously monitor the fluorescently labeled DNA and/or DNA-bound proteins (Supplementary Figure S4). Unlike previously used single-molecule assays that involve a free DNA end due to single-end tethered DNA molecule and uneven forces on DNA (12,25,46), our experimental configuration contains no free DNA ends, and the force applied on DNA is uniform.

**Figure 2.**
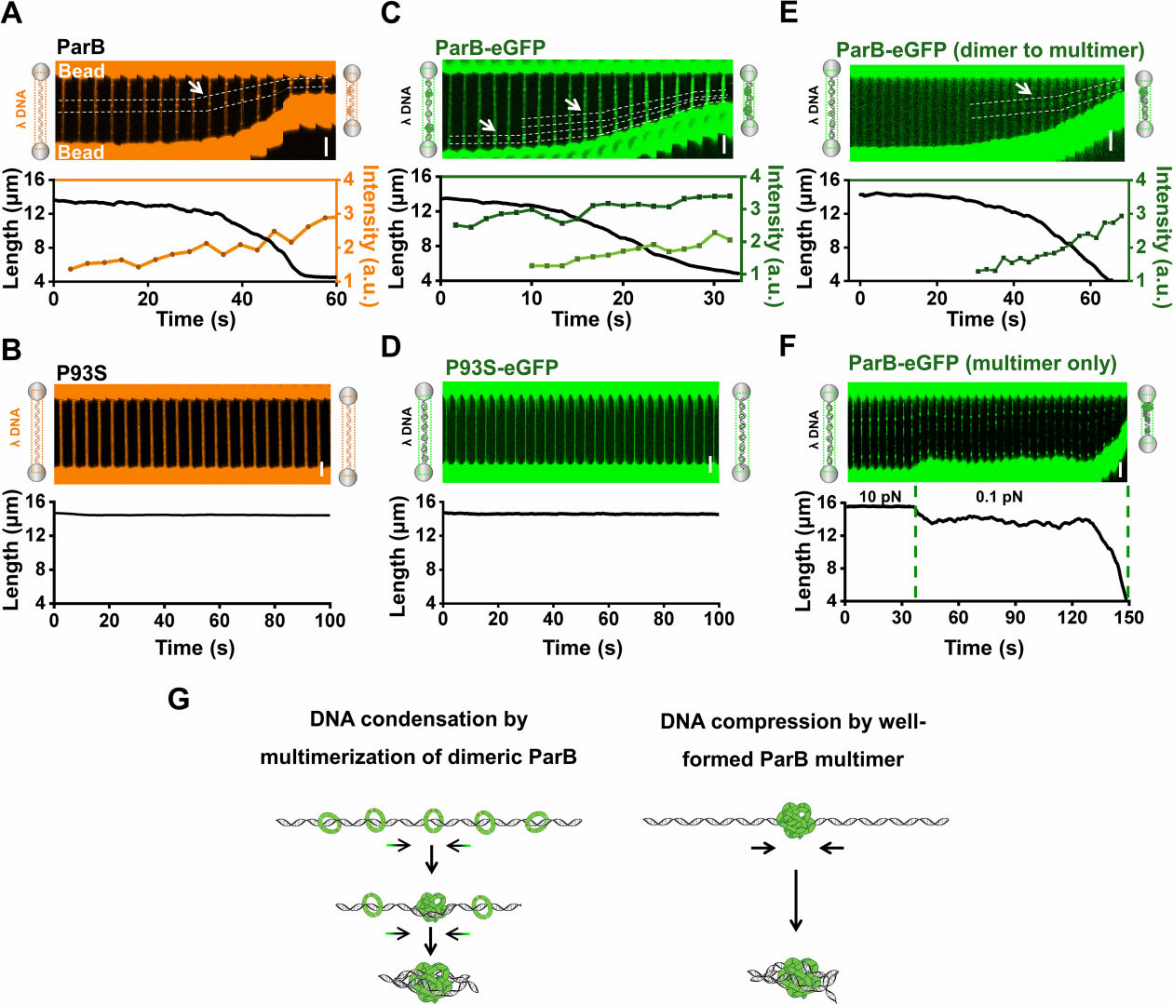
Single-molecule measurements of DNA condensation by ParB. (A and B) A representative kymograph showing the λ DNA (orange) in the presence of 250 nM ParB (**A**) or P93S (**B**) and 200 nM SYTOX-Orange under 0.1 pN. The corresponding DNA length (black) and the intensity of the DNA foci (orange) are shown below the kymographs. The dotted white line highlights the area of DNA ‘foci’. (C and D) A representative kymograph showing the fluorescence signal of ParB-eGFP (**C**) or P93S-eGFP (**D**) along the λ DNA under 0.1 pN. The corresponding DNA length (black) and the ParB-eGFP multimers (green) intensity are shown below the kymograph. The white arrows indicate the preformed and gradually formed ParB multimers. (**E**) A representative kymograph showing the fluorescence signal of ParB-eGFP (250 nM) along the λ DNA under 0.1 pN. A white arrow indicates that a single ParB multimer is gradually formed along with the DNA shorting. The corresponding DNA length (black) and the intensity of the ParB multimers (green) are shown below the kymograph. (**F**) A representative kymograph illustrates the fluorescence of ParB-eGFP along the λ DNA under 10 pN and 0.1 pN as indicated by two green dashed lines. The corresponding DNA length is shown below the kymograph. (**G**) Two DNA compaction modes enabled by phase-separated ParB multimers. The colored arrows indicate the co-condensation of DNA and ParB. The black arrows indicate the DNA condensation.

We first conducted the DNA condensation assay with a 48.5-kbp λ DNA molecule containing no *parS* motifs. According to previous studies, a high protein concentration is required for ParB to condense nsDNA (46–48). We thus examined the suspended DNA molecule in a 250 nM ParB protein channel, wherein a high-frequency feedback system was employed to maintain a constant force on the DNA. Indeed, DNA condensation occurred more frequently when subjected to a force lower than 3 pN (Supplementary Figure S5). Accordingly, we used a force of 0.1 pN in the following assays to ensure DNA condensation unless otherwise stated. To directly visualize DNA status, we incorporated 200 nM SYTOX-Orange into the protein channel as a probe for dsDNA (49). As indicated by the gradual decrease in DNA length, DNA condensation also occurred in the presence of SYTOX-Orange (Figure 2A). Meanwhile, the fluorescence imaging reflected the emergence of a few ‘foci’ along the DNA (Figure 2A). Notably, these ‘foci’ became more pronounced along with the gradual decrease in DNA length, suggesting a random local DNA coacervation as a reason for DNA shortening. In stark contrast to wild-type ParB, P93S failed to condense all examined DNA tethers DNA (*n* = 12) under the same condition (Figure 2B). Control experiments excluded the possibility that the DNA binding ability of P93S was abolished (Supplementary Figure S6).

To better understand how ParB induces DNA condensation, we used the ParB-eGFP protein in the DNA condensation assay to visualize DNA-bound proteins directly. As expected, ParB-eGFP at high concentrations was found to condense DNA against a low force. Analysis of the fluorescence signals enabled us to determine the multimeric status of ParB (Supplementary Figure S7). During DNA condensation, we observed the ParB-eGFP multimers that were either preformed or formed concurrently with DNA shortening (Figure 2C). Consistently, the majority of P93S-eGFP was monitored to uniformly bind the suspended DNA in a dimeric form for up to 5 minutes, lacking the ability to condense DNA, and its multimeric form was scarcely observed (Figure 2D and Supplementary Figure S7). Along a few condensed DNA molecules by ParB-eGFP, DNA condensation was accompanied by a single gradually formed ParB multimer, wherein no preformed ParB-eGFP multimers existed (Figure 2E). It is noteworthy that within these DNA molecules, the increase in the fluorescence intensity of the signal ParB multimer is correlated with the decrease in the DNA length, favoring that ParB multimerization induces DNA condensation (Figure 2E). In support of this notion, stretching the DNA tether right after the initial DNA condensation can reverse the multimerization of ParB (Supplementary Figure S8). Combined with the findings with the fluorescently labeled DNA, these data support that the gradual ParB multimerization drives the co-condensation of protein-bound DNA.

We next asked if the well-established ParB multimers could promote DNA condensation. To avoid the effect of ParB dimer on DNA condensation, we transported a ParB-bound DNA molecule to a protein-free channel. In this channel, a force of 10 pN was applied for approximately 1 minute and 30 seconds to prevent DNA condensation and to dissociate ParB dimers from the DNA, while the multimeric ParB remained bound to DNA (Supplementary Figure S9). After lowering the force to 0.1 pN, 44% of the examined ParB multimer-bound DNA molecules (*n* = 16) gradually shortened (Figure 2F). Since newly formed ParB multimers were undetectable during DNA condensation, these data suggest that well-established ParB multimers indeed have the capability to condense DNA. DNA condensation in this scenario likely occurs as ParB multimers pulled adjacent DNA into the coalesced structures. Moreover, DNA-bound ParB-eGFP multimers can fuse and recover fluorescence signals after photobleaching, suggesting that these micro-condensates exist in a phase-separated state (Supplementary Figure S10).

In summary, our study uncovers two distinct mechanisms by which ParB multimers condense DNA. The DNA can either be condensed through the gradual aggregation of DNA-bound ParB dimer or be compressed into pre-existing ParB condensates (Figure 2G).

### Phase-separated ParB multimer loops distant DNA and stabilizes the nucleoprotein complex

**Figure 3.**
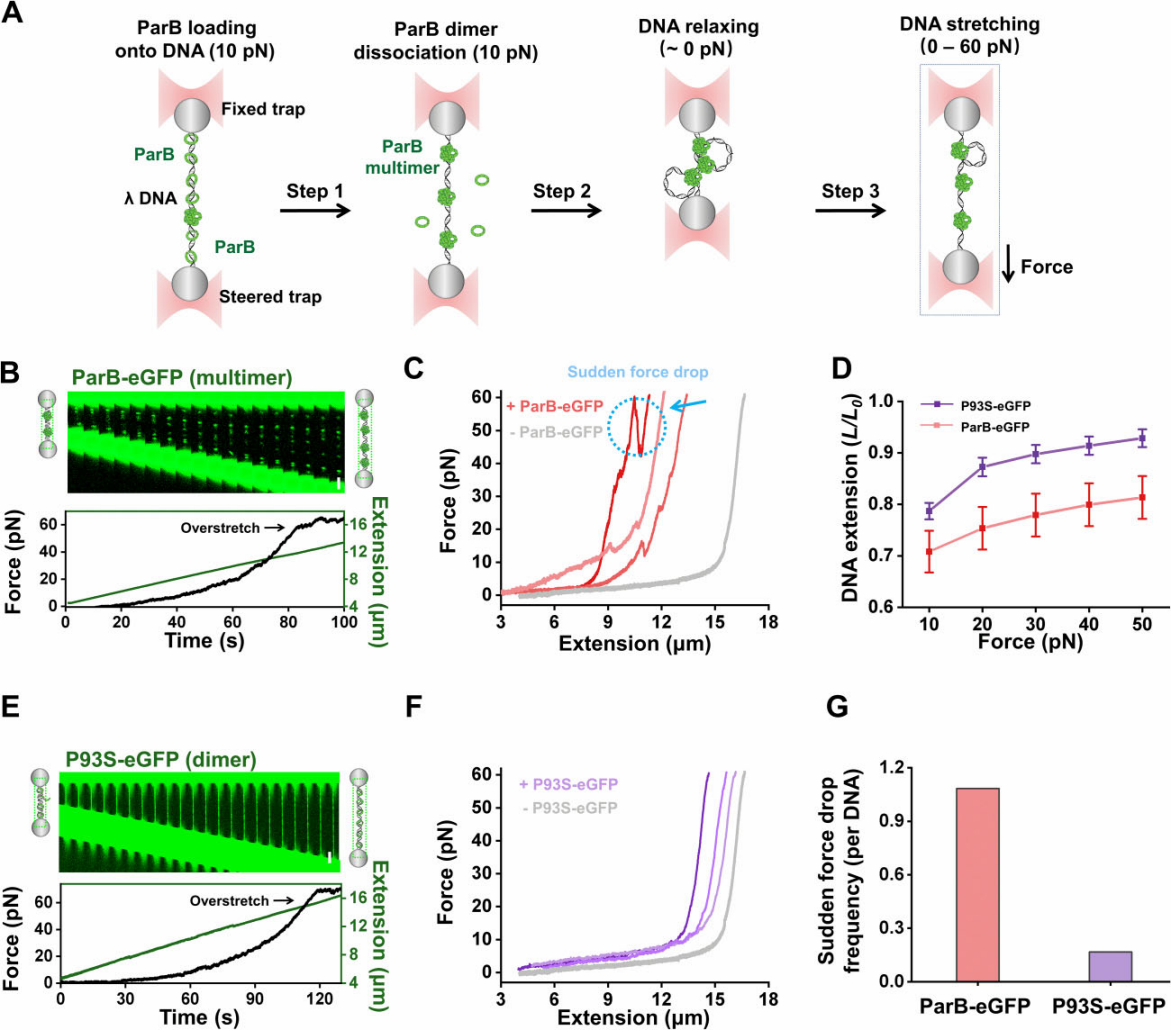
Stretching of ParB-bound relaxed DNA. (**A**) Cartoons illustrating the experimental procedures. After loading ParB proteins on a single λ DNA, it is transported to the buffer channel to dissociate dimeric ParB. Then, the microsphere in the steered trap is actively moved to the fixed trap for DNA relaxation. ParB-bound relaxed DNA is stretched by moving the steered trap away from the fixed trap at 0.1 μm/s. (**B**) A representative kymograph of a λ DNA showing the stretching of DNA bound by ParB multimers. The corresponding force and extension of the DNA are shown below the kymograph. (**C**) Representative force–extension curves of DNA bound by ParB multimers only. The grey line represents the standard force–extension curve of λ DNA in the absence of ParB. A sudden force-dropping event is highlighted in a dotted blue circle. (**D**) The normalized DNA extension (*L/L_0_*) under different forces with ParB multimer binding or P93S dimer binding. *L_0_* is the original length of naked λ DNA. The data are shown in mean ± SEM. *n* = 14 and 15. (**E**) A representative kymograph of a λ DNA showing the stretching of DNA bound by P93S dimers. The corresponding force and DNA extension are shown below the kymograph. (**F**) Representative force–extension curves of DNA bound by P93S dimer. The grey line represents the standard force–extension curve of λ DNA in the absence of P93S-eGFP. (**G**) The frequency of the sudden drop in force detected in the force–extension curves.

Our experiments have demonstrated the capability of multimerized ParB in condensing DNA against a force of 0.1 pN, under which the DNA was stretched to about 85% of its contour length. Under this condition, DNA-bound ParB can only access adjacent DNA for compaction. Our previous study has shown that ParB multimers can bridge two dsDNA molecules (30). It is posited that in addition to condensing nearby DNA, ParB multimers might also loop and compact distant DNA segments. To explore this possibility, we developed a DNA relaxation and stretching assay (Figure 3A). Specifically, after ensuring the attachment of ParB multimers to DNA in the optical tweezers assay, we relaxed the DNA by bringing the two microspheres approximately 4 μm away for 5 seconds. This relaxation state of DNA (∼0 pN) allows DNA-bound ParB multimers to gain access to distant DNA segments. Subsequently, we stretched the DNA by moving one microsphere away from the other at a constant rate of 0.1 μm/s. We then compared the force–extension curves of ParB multimer-bound DNA to those without ParB.

Since SYTOX-Orange is an intercalated fluorescent dye that can influence the mechanical response of DNA molecules (49), we chose to use ParB-eGFP in this assay for fluorescence imaging. Kymograph analysis showed that ParB multimers remained bound to DNA during stretching, even when the force ramped up to approximately 60 pN (Figure 3B). The consistent fluorescence signals from the ParB multimers during DNA stretching could be attributed to their dynamically arrested micro-condensate state. Meanwhile, the obtained force–extension curves conformed to the worm-like chain (WLC) model with apparently shortened contour lengths (50) (Figure 3C and Supplementary Figure S11). Upon verifying that the mechanical elasticity of DNA bound by ParB remained unchanged within a force range of 10–60 pN (Supplementary Figure S12), we concluded that the relaxed DNA (∼0 pN) was compacted by preformed ParB multimers and that increasing the force to 60 pN could not fully revert the DNA to its original length. We chose not to examine the sustainability of the nucleoprotein complex beyond 60 pN, as the altered double-helix structure of DNA due to overstretching may impair ParB binding (37). Notably, almost all the force–extension curves exhibited frequent sudden drops in force (Figure 3C). Statistical analysis reveals that DNA length increases by hundreds of nanometers (Supplementary Figure S11), supporting a DNA looping mechanism facilitated by ParB multimers. To provide direct evidence for ParB multimer-mediated DNA looping, a vertical flow was applied to the shortened DNA tethers to straighten the potentially looped DNA for direct fluorescence monitoring. As expected, DNA loops were visualized under the flow, with ParB multimers located near the DNA junctions. (Supplementary Figure S13).

To address whether the ability to loop DNA is unique to ParB multimers, we revisited this experiment by replacing the ParB-eGFP with P93S-eGFP, which is supposed to bind to dsDNA mostly in a dimeric form rather than a multimeric form. During DNA stretching following relaxation, we consistently observed a uniform coating of dimeric P93S-eGFP along the DNA, with minimal evidence of protein multimerization (Figure 3E). In line with the findings with P93S on tensioned DNA, it also exhibited a weakened ability to form multimers on a relaxed DNA molecule. Moreover, the force–extension curves of P93S-eGFP-bound DNA closely resembled those of naked DNA, indicating that the DNA in the relaxing state was only slightly condensed by dimeric P93S-eGFP (Figure 3F–G). Moreover, these curves are smoother, with a significant reduction in sudden force-dropping events (Figure 3D–E). Experiments with ParB-ΔNTD-eGFP showed similar observations (Supplementary Figure S14). Thus, the sudden drop in force detected with ParB-eGFP is likely attributable to the ParB multimer.

In conclusion, ParB multimers on DNA can loop distant DNA segments. The ParB multimer-involved nucleoprotein complex forms an exceptionally stable structure, able to withstand disruptive forces of tens of piconewtons.

### CTP and *parS* promote the formation of phase-separated ParB for two-step DNA condensation

**Figure 4.**
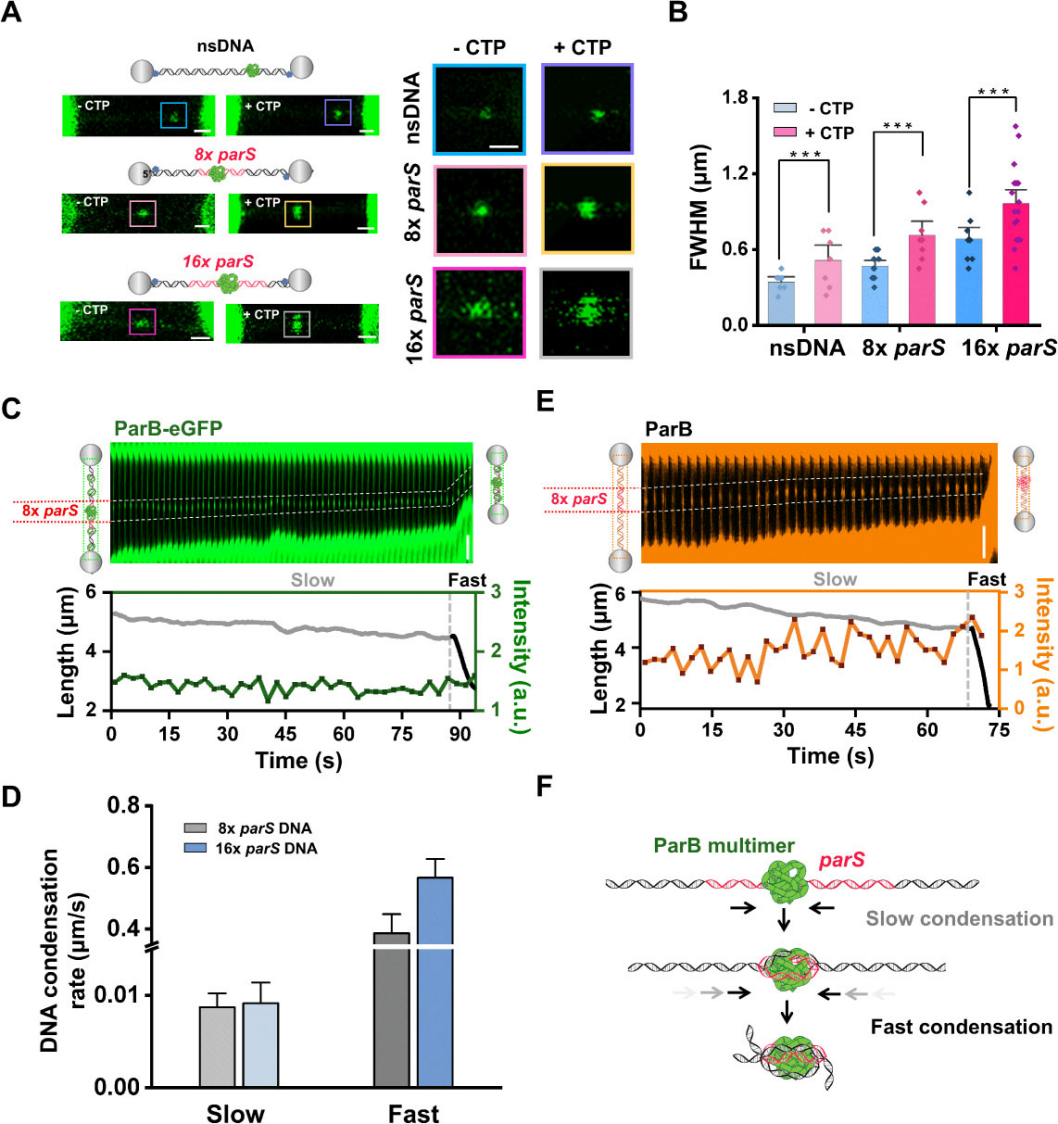
CTP and *parS* motifs regulate ParB condensate formation for two-step DNA condensation. (**A**) Left: Schematics of the 0×, 8× and 16× *parS* DNA templates and confocal images of ParB multimers binding to the templates in the absence and presence of 2 mM CTP. Right: Zoom-in images of ParB multimers bound to the templates. Scale bar, 1 μm. (**B**) Statistical analysis of the size of the ParB multimer under indicated conditions. FWHM, full width at half maximum. Data are presented as mean ± SEM. *n* = 7, 10, 8, 9, 11 and 16, respectively. ***P < 0.001. (**C**) A representative kymograph of the 8x *parS* DNA showing the ParB-eGFP (50 nM) signal under 0.1 pN and 2 mM CTP. The corresponding DNA length (gray for slow DNA condensation and black for fast DNA condensation) and the intensity of the ParB-eGFP multimer (green) are shown below the kymograph. The dotted white line highlights the ParB multimer at the *parS* sites. Scale bar, 2 μm. (**D**) The fast and slow DNA condensation rates (mean ± SEM) mediated by ParB on the 8x and 16× *parS*-containing DNA templates in the presence of 2 mM CTP. (**E**) A representative kymograph of the 8× *parS*-containing DNA showing the SYTOX-Orange-stained DNA in the presence of 50 nM ParB under 0.1 pN. The corresponding DNA length (gray for slow DNA condensation and black for fast DNA condensation) and the DNA condensate intensity (orange) are shown below the kymograph. The dotted white line highlights coacervated DNA at the *parS* motifs selected for the fluorescence intensity measurements. Scale bar, 2 μm. (**F**) The model of two-step DNA condensation mediated by the *parS*-bound ParB condensate.

Given that ParB is a CTPase with a binding preference for *parS* (19,20), we next explored how CTP and *parS* influence DNA condensation by phase-separated ParB multimers. We constructed three 27-kbp DNA templates that contain 0x, 8x, and 16x *parS* motifs, respectively (Supplementary Table S1). The *parS* motifs are located right in the middle of the DNA templates (Supplementary Figure S1). We first examined the formation of ParB multimers on these DNA templates. Using optical tweezers, we applied a constant force of 10 pN on these DNA templates to prevent DNA condensation while monitoring the fluorescence signal of DNA-bound ParB proteins. In agreement with our previous study (30), ParB-eGFP preferred forming a single multimer at *parS* motifs even under a low protein concentration of 50 nM (Figure 4A). The sizes of these ParB multimers are generally larger at the *parS* motifs compared to those on nsDNA, and they increased with the number of *parS* motifs (Figure 4A–B). This indicates that *parS* motifs promote the coacervation of ParB-eGFP proteins along DNA. Moreover, the addition of CTP further stimulates ParB multimerization, as seen by the increase in the multimer size (Figure 4A, B). Therefore, both *parS* and CTP promote the formation of ParB multimers on DNA.

We next asked whether a single ParB multimer at *parS* motifs is sufficient to trigger DNA condensation. We maintained a low force of 0.1 pN on the 8x *parS*-containing DNA template. Up to 80% of the DNA molecules were found to be condensed in the 50 nM ParB channel (*n* = 17). In stark contrast, none of the 20 DNA molecules lacking *parS* motifs condensed under the same experimental condition (Supplementary Figure S15). The presence of *parS* motifs allows DNA condensation under a low ParB concentration, highlighting their importance in partition complex assembly. Interestingly, 87% of the examined 8x DNA molecules (*n* = 15) initially shortened at an extremely low rate (∼0.008 μm/s), followed by a rapid condensation rate (∼0.38 μm/s) (Figure 4C–E and Supplementary Figure S16). A similar two-step DNA condensation pattern was observed in 12 out of the 13 examined 16x *parS* DNA tethers (Supplementary Figure S17). Kymograph analysis revealed a single ParB multimer at *parS*, persisting throughout the DNA condensation process without apparent expansion. Therefore, it is highly likely that the single ParB multimer at *parS* could attract nearby DNA for compaction. To verify this, we used SYTOX-Orange to directly visualize DNA condensation by ParB. The DNA molecules consistently exhibited two-step condensation in the presence of SYTOX-Orange (Figure 4E). Importantly, coacervated DNA appeared and slowly grew around the *parS* motifs during the slow DNA shorting process. Therefore, it is conceivable that the DNA molecule was condensed by ParB multimers at *parS* sites. The subtle increase in fluorescence intensity of DNA at *parS* motifs during condensation is attributed to the low compaction ratio in the slow condensation regime (Figure 4E and Supplementary Figure S16B). Unfortunately, the subsequent rapid DNA shortening phase occurred too swiftly to capture fluorescence changes.

In summary, a single ParB multimer, maintaining a micro-condensate state at the *parS* motifs, can compress DNA into the condensate in a two-step manner (Figure 4F). The rapid DNA condensation is likely triggered by DNA accumulation within these condensates.

### *ParS*-centered ParB–DNA co-condensate maintains a stable nucleoprotein architecture

**Figure 5.**
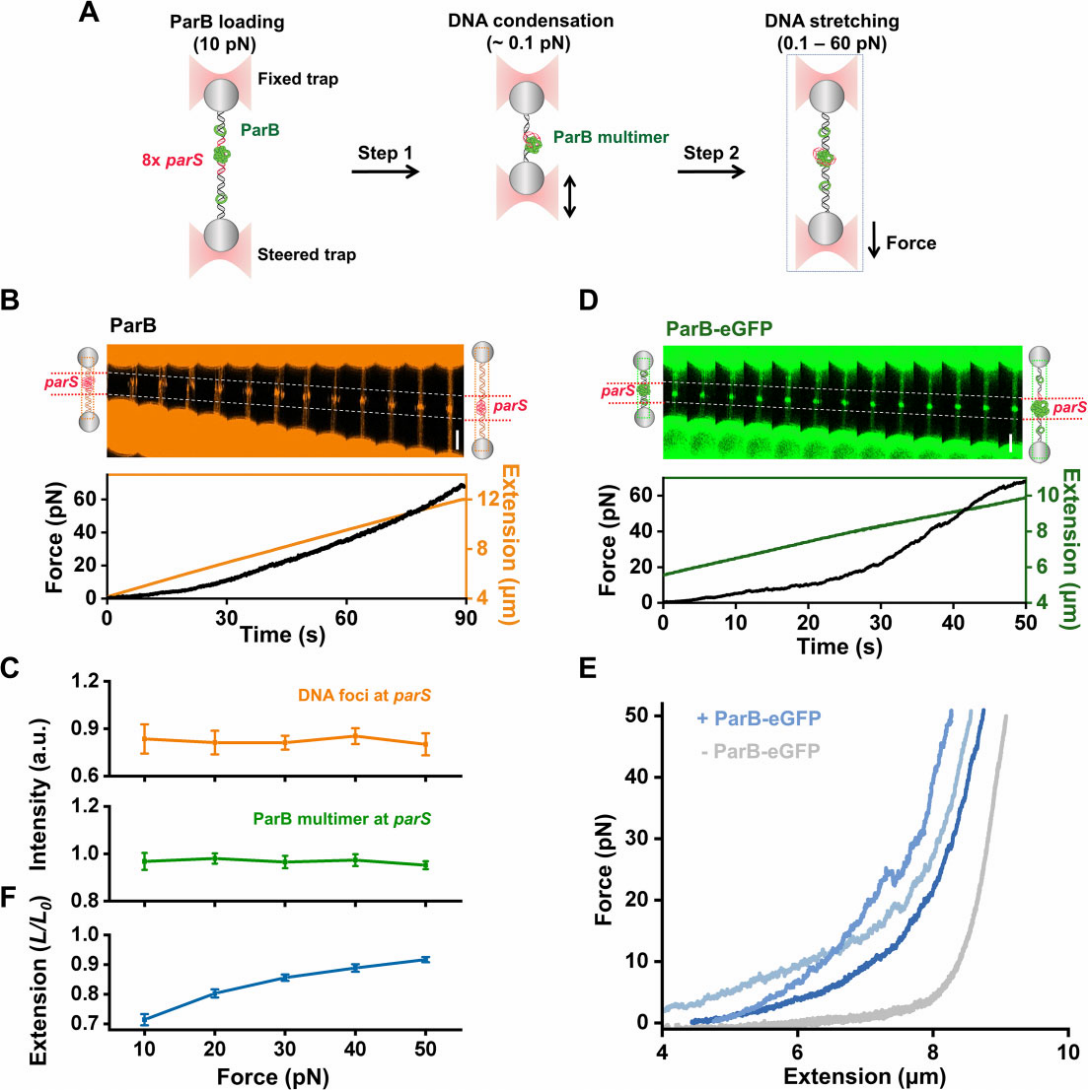
Stretching of the DNA condensed by the *parS*-bound ParB condensate. (**A**) A cartoon illustration of the experimental configuration. After the phase-separated ParB condensate binding to *parS* and condensing DNA under 0.1 pN, the 8× *parS*-containing DNA is stretched by moving the steered trap away from the fixed trap at 0.1 μm/s. (**B**) A representative kymograph of the 8× *parS*-containing DNA showing the DNA stretching after condensation in the presence of 2 mM CTP by the *parS*-centered ParB condensate. The corresponding DNA length (orange) and the force (black) are shown below the kymograph. The dotted white line highlights the DNA condensate at the *parS* motifs selected for the fluorescence intensity measurements. Scale bar, 2 μm. (**C**) The intensity of ParB multimer (green) and DNA foci (orange) at the *parS* sites under different forces during DNA stretching in the presence of 2 mM CTP (n = 11 and 10, respectively). (**D**) A representative kymograph of the 8× *parS* DNA showing the ParB-eGFP signal during DNA stretching in the presence of 2 mM CTP. The corresponding DNA length (green) and the force (black) are shown below the kymograph. The dotted white line highlights the ParB multimer at the *parS* sites. Scale bar, 2 μm. (**E**) Representative force–extension curves of the 8x *parS* DNA in protein channel with 50 nM ParB-eGFP and 2 mM CTP. The grey curve is the standard force-extension relationship of the naked 8× *parS* DNA. (**F**) The relative DNA extension (*L/L*_0_) (mean ± SEM) under different forces with ParB-eGFP binding at the *parS* motifs in the presence of 2 mM CTP (*n* = 10). *L*_0_ is the original length of naked λ DNA.

Since nsDNA condensed by phase-separated ParB multimers can withstand significant disruptive forces (Figure 3C), the co-condensate created by ParB and DNA at *parS* sites is likely a stable nucleoprotein structure. We further examined the stability of the co-condensate of ParB and DNA at *parS*. After the 8x *parS*-containing DNA was condensed by *parS*-located ParB condensate, the DNA tether was stretched at 0.1 μm/s in the presence of SYTOX-Orange. Coacervated DNA at *parS* was immediately observed throughout the DNA stretching process (Figure 5A, B). Additionally, statistical analysis showed that the fluorescence signal of the stretched DNA remained relatively constant within a force range of 10–50 pN (Figure 5C). This unchanging fluorescence signal indicates that the condensed DNA at *parS* motifs did not revert to a linear state under increased force, maintaining its condensed form. Meanwhile, DNA stretching experiments with ParB-eGFP also demonstrated the existence of ParB micro-condensate at the *parS* motifs even after DNA overstretching (Figure 5D). The fluorescence intensity of the condensates remained fairly consistent under varying forces, indicating the stability of the ParB condensates during DNA stretching (Figure 5C). The continuous presence of coacervated DNA and ParB condensates at *parS* motifs, along with the steady fluorescence signals during DNA stretching, strongly supports the durability of the *parS*-centered ParB–DNA co-condensate. This notion is further corroborated by analyzing the force–extension curves of the DNA condensed by *parS*-centered ParB-eGFP condensates (Figure 5E). Despite the DNA approaching its natural length under increased force, about 10% of the examined DNA molecule remained inextensible even under forces up to 50 pN (Figure 5F), indicating persistent DNA condensation. Compared to λ DNA, the low condensation ratio observed with *parS* DNA can be attributed to the limited formation of ParB multimers under low protein concentrations. (Figures 3D and 5F). Therefore, the *parS*-centered ParB–DNA co-condensate is a stable entity, likely contributing to the preservation of the partition complex's integrity. Moreover, evidence supporting DNA looping by the *parS*-centered phase-separated ParB comes from the observed sudden drop in the force–extension curves of the *parS*-containing DNA (Supplementary Figure S18).

## Discussion

In ParAB*S*-mediated bacterial chromosome segregation, effective chromosomal DNA condensation and compaction mediated by ParB molecules are imperative for assembling the partition complex (3,31). In particular, ParB undergoes dimerization and multimerization to confer specificities and functional advantages for DNA organization (12,25,45,51). The nucleoprotein assembly begins with dimeric ParB binding to *parS* sites and spreading to neighboring DNA (7). In this work, we filled the knowledge gap concerning how ParB dimers further compact DNA into a network of the DNA–protein complex.

In line with previous studies (28,29), BsParB can also undergo phase separation (Figure 1A). Compared with ParB from the *F* plasmid and *C. glutamicum*, BsParB condensates exhibited a notably slower FRAP recovery, indicating a less dynamic property. (25,28,29). This slower FRAP recovery could be attributed to differences in the mechanism of phase separation between different proteins and different conditions to initiate condensation (Figure 1B). The presence of DNA leads to the co-condensation of ParB and DNA (Figure 1C). In contrast to nsDNA, the *parS*-containing DNA segment further potentiates the co-condensation of ParB with DNA under a low protein concentration (Figure 1E). The regulation of protein phase separation by specific DNA sequences has been reported on other proteins, such as the transcription factors p53 and FoxA1 (52,53). These similar observations can be rationalized by the high affinity of protein to the binding sites, providing local specificity and sequence-specific nucleation. Notably, the NTD of ParB responsible for multimerization is also indispensable for phase separation (Figure 1A). It is not surprising that key residues of ParB for multimerization overlap with those for phase separation, as multimerization is a prerequisite for phase separation. An *in vivo* study has reported that a chromosome segregation defect can arise from a single amino acid mutation in the NTD of ParB (33). It is tempting to speculate that ParB’s impaired phase separation ability is one of the reasons for the defective segregation. However, our findings do not necessarily exclude the involvement of the CTD of ParB in the phase separation as this domain is mainly in charge of ParB dimerization, a prerequisite for multimerization (51).

After demonstrating the phase separation ability of ParB, we further examined the potential functional roles of phase separation of ParB in DNA organization. The phase-separated ParB multimer can compel diverse DNA compaction and condensation modes. First, the tendency of DNA-bound ParB molecules to aggregate generates a pulling force on DNA for co-condensation (Figure 2E). Second, well-established ParB condensates can further condense the DNA by compressing the adjacent DNA into the micro-condensate (Figure 2F). Third, in addition to adjacent DNA, phase-separated ParB can loop distant DNA (Figure 3B–C). DNA-bound protein condensates driving the growth of the phase by pulling non-condensed DNA have been observed with other proteins, such as the transcription factor FoxA1 and the BLM helicase (53,54). Although the DNA pulling force generated by ParB phase separation is not significant (less than 1 pN), it is an irreversible process as applying a stretching force of tens of piconewtons on the ParB-condensed DNA cannot completely disassemble the co-condensates (Figure 3C, D). Similarly, other works have also reported the irreversibility of ParB-condensed DNA (26,55). In stark contrast, the condensed DNA by the P93S variant is relatively weak, which can be largely reversed by a stretching force (Figure 3D–F). Therefore, it is conceivable that phase-separated ParB multimers stabilize the partition complex while ParB dimers confer transient interactions.

**Figure 6.**
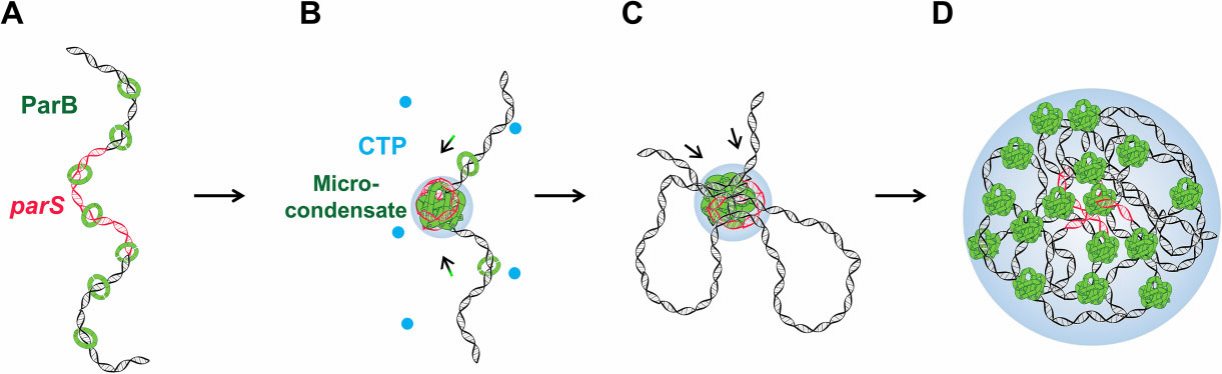
A proposed model of phase-separated ParB seeding the partition complex assembly. During the partition complex assembly, ParB dimers are initially loaded onto *parS* motifs and spread to neighboring DNA (**A**). The phase separation ability, promoted by CTP, allows the ParB proteins to aggregate and form condensates around the *parS* sites (**B**). The DNA condensation accompanies the formation of ParB condensates along the DNA. The ParB–DNA co-condensates can further compact the DNA by compressing neighboring DNA (indicated by the colored arrows) or looping distant DNA (**C**). The *parS*-bound micro-condensates of ParB and DNA may seed the following assembly of the macro-condensate of ParB and DNA (**D**). The *parS*-centered partition complex is ensured by the immobilization of ParB condensates at the *parS* sites. The colored arrows indicate the co-condensation of DNA and ParB. The black arrows indicate the movement of DNA into the condensate.

Our work also explains the decrease in ParB concentration required for DNA condensation due to the presence of *parS* and CTP and the architecture of the *parS*-centered complex (25,26). During the partition complex assembly, the DNA adjacent to *parS* is first condensed along with the ParB dimers' aggregation, forming ParB–DNA co-condensates. According to our previous study (30), tens of ParB molecules around the *parS* sites are sufficient for initiating DNA–protein assembly. CTP promotes ParB binding around *parS* sites and subsequent ParB clustering for condensate formation (Figure 4A–B) (30–32). Importantly, the assembled ParB–DNA condensates at the *parS* sites prefer to remain immobile and allow further DNA compaction by bringing adjacent DNA into the condensate and bridging distant DNA (Figure S13) (24,30). This feature ensures *parS*-centered DNA assembly. The *parS*-centered ParB–DNA co-condensates remain in a stable structure and possibly seed the following assembly of the partition complex. Interestingly, the condensation of *parS*-containing DNA proceeded in a two-step manner (Figure 4C–E). The fast DNA condensation process could be due to the accumulated DNA in the co-condensates. The gradually enlarged co-condensate may further stimulate DNA compression. Whether the DNA condensation ability depends on the size of the ParB–DNA co-condensate warrants further exploration. We summarized our major findings by proposing a refined model of phase-separated ParB functioning in assembling the higher-order nucleoprotein complex (Figure 6). Overall, our work highlights the importance of the phase separation ability of ParB in DNA condensation and compaction and sheds new light on the molecular mechanism underlying the assembly and maintenance of the bacterial partition complex.

## Supplementary Material

gkae533_Supplemental_File

## Data Availability

All data are available from the corresponding authors upon reasonable request and/or included in the manuscript as figure source data or supplementary data.
