# Supplementary material for: Phase-separated ParB enforces diverse DNA compaction modes and stabilizes the parS-centered partition complex
Source: Nucleic Acids Res. 2024 Jun 22;52(14):8385–98. doi: 10.1093/nar/gkae533 (PMC11317135; doi:10.1093/nar/gkae533)
Supplement: gkae533_Supplemental_File [file gkae533_supplemental_file.pdf]

Supplementary Materials

Phase-separated ParB enforces diverse DNA compaction modes and stabilizes the *parS*-centered partition complex

Yilin Zhao[1,†], Lijuan Guo[1,†], Jiaojiao Hu[2,3†], Zhiyun Ren[1,4,5], Yanan Li[1], Meng Hu[1], Xia Zhang[1], Lulu Bi[1], Dan Li[6,7], Hanhui Ma[1], Cong Liu[2,3,*], and Bo Sun[1,*]

*To whom correspondence should be addressed. Tel: +86 21 2068 4536; Fax: +86 21 2068 5430; Email: sunbo@shanghaitech.edu.cn

Correspondence may also be addressed to Cong Liu. Tel: +86 21 6858 2528; Fax: +86 21 6858 2528; Email: liulab@sioc.ac.cn

This file includes

Supplementary Figures S1 – S18

Supplementary Table S1

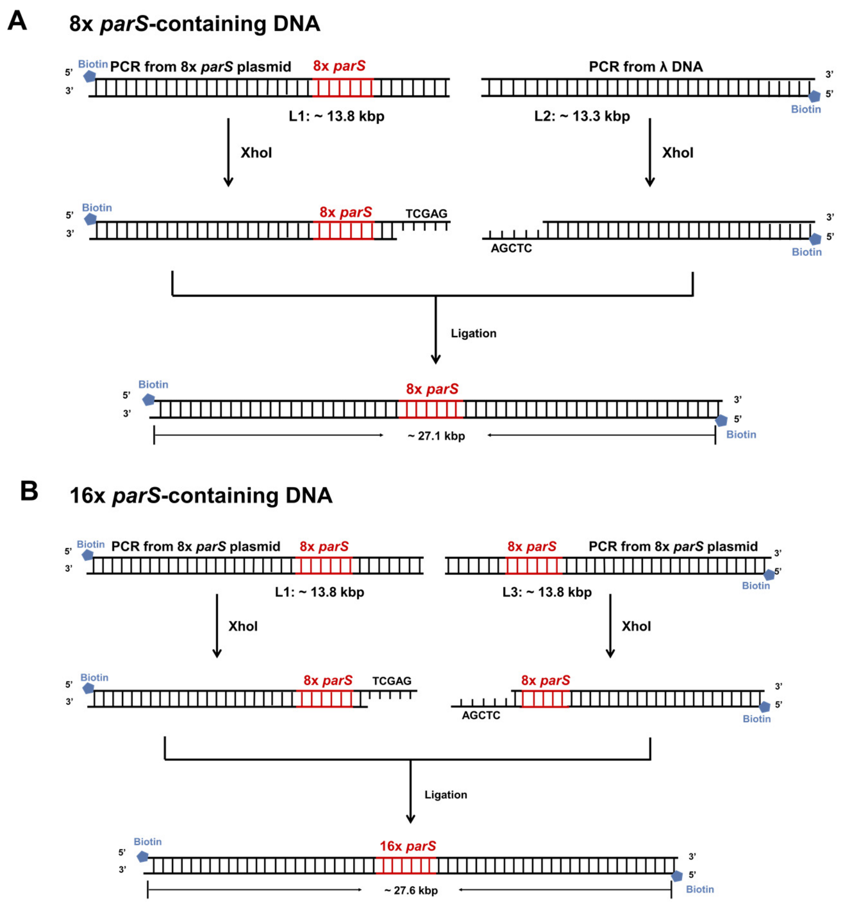

Supplementary Figure S1. Construction of the 8x and 16x *parS*-containing DNA templates. A. The DNA template containing an 8x *parS* motif was the ligation product of two DNA segments, L1 and L2. **B.** The 16x *parS* template was similarly prepared using L1 and L3. The 13.8-kbp L1 and L3 DNA segments were PCR-amplified from the 13.8-kbp plasmid containing 8x *parS*. The 13.3-kbp L2 was PCR-amplified from λ DNA. The resulting DNA fragments were digested with XhoI (NEB) to create an overhang for ligation. The 8x *parS*-containing DNA sequence is shown below with *parS* motifs colored red.

5'-…**TGTTACACGTGAAACACGCACTCAGCTTGACATGGCGCTAAATATCGGCGTTTCACGTGAAACA CACCGTGTGGAATACACCCGGCATCGCGTCCTTGTTACACGTGAAACAGCCATCCAGATTGTACTCA CCGATGGACACACTGTTACACGTGAAACACCCTTGGCAGGGTCAACAGCACCTTGGGATTGGAATT CGCGGCCGCCGCGACCGACGTGTGCTGTTACACGTGAAACACGCACTCAGCTTGACATGGCGCTAA ATATCGGCGTTTCACGTGAAACACACCGTGTGGAATACACCCGGCATCGCGTCCTTGTTACACGTGA AACAGCCATCCAGATTGTACTCACCGATGGACACACTGTTACACGTGAAACA**…-3'

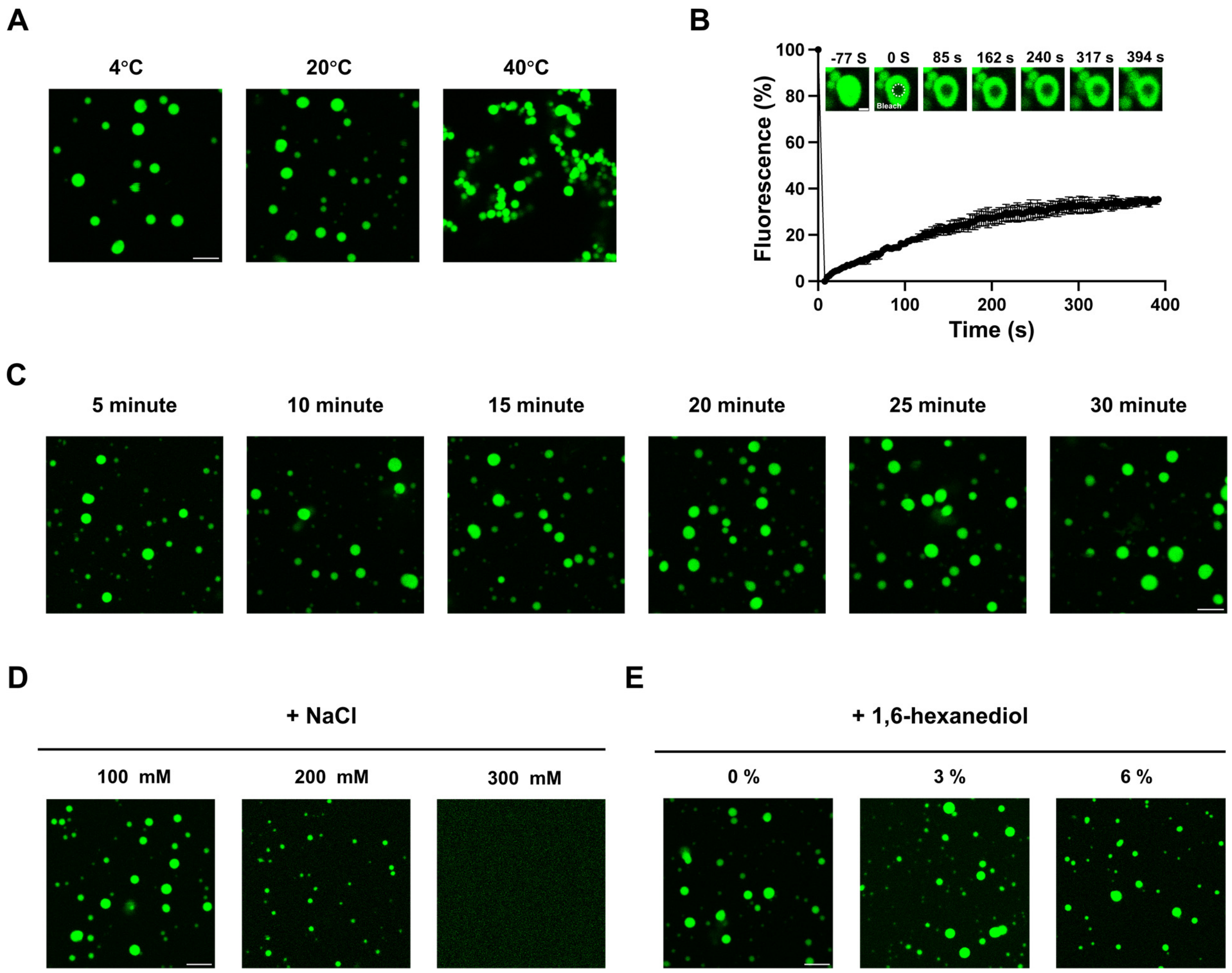

Supplementary Figure S2. ParB condensation under varied experimental conditions. A. Representative confocal images of ParB (20 μM) condensation in buffer containing 50 mM Tris-HCl pH 7.5 and 100 mM NaCl at different temperatures. **B.** Fluorescence recovery curves for ParB condensates formed at 40°C, with values shown as mean ± SD from three replicates. **C**. Representative confocal images of ParB (20 μM) condensation in buffer containing 50 mM Tris-HCl pH 7.5 and 100 mM NaCl at different time points. **D**. Representative confocal images of ParB condensation in buffer containing 50 mM Tris-HCl, pH 7.5 and indicated concentration of NaCl. **E**. Representative confocal images of ParB condensation with the incorporation of 1,6-hexanediol in buffer containing 50 mM Tris-HCl, pH 7.5 and 100 mM NaCl. Scale bar, 5 μm.

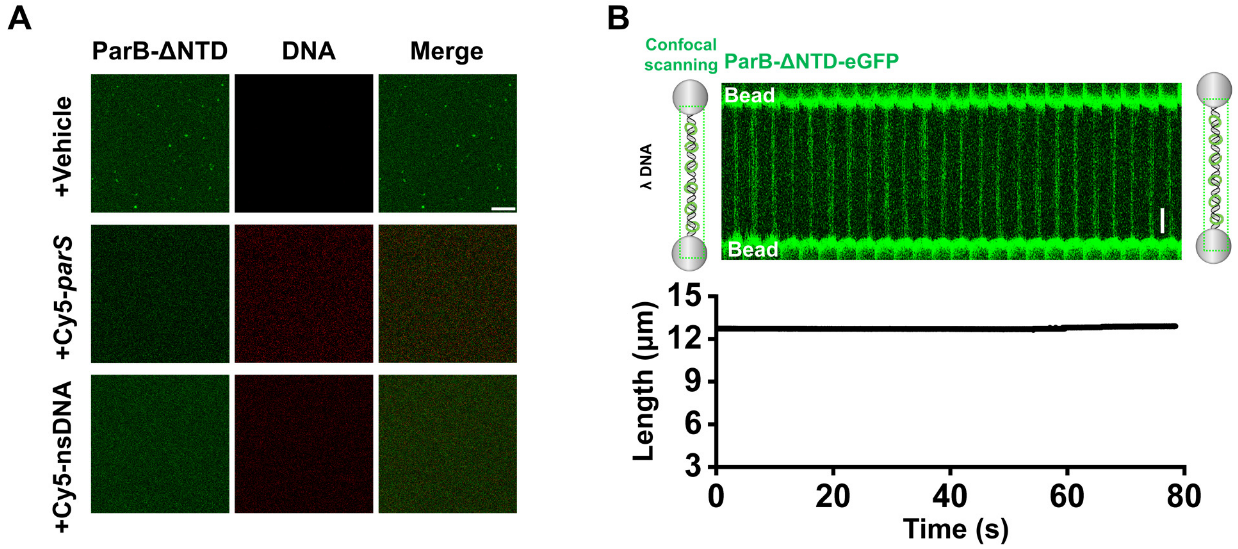

Supplementary Figure S3. Phase separation and DNA condensation capability of ParB-ΔNTD. A. Co-condensation images of 20 μM ParB-ΔNTD with 1 μM Cy5-*parS* or Cy5-nsDNA. Scale bar, 5 μm. **B.** A representative kymograph showing the fluorescence signal of ParB-ΔNTD (250 nM) along the λ DNA under 0.1 pN. The corresponding DNA length is shown below the kymograph. Scale bar, 3 μm.

ParB-ΔNTD cannot phase-separate with or condense DNA.

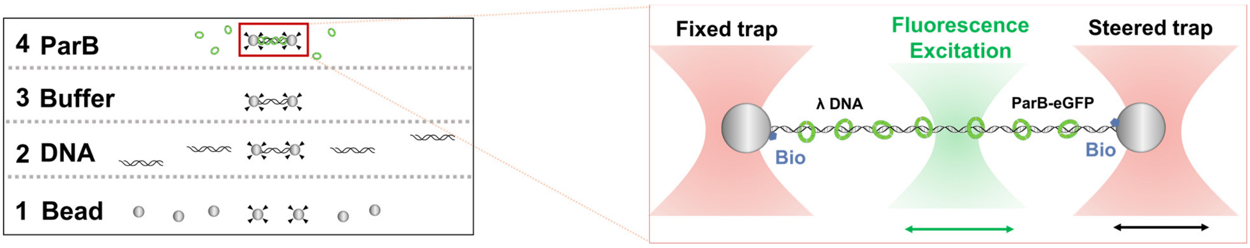

Supplementary Figure S4. Schematic of the experimental setup and configuration. A single DNA tether is formed in Channels 1 – 3, containing streptavidin-coated beads, biotinylated λ DNA, and reaction buffer, respectively. The successfully formed DNA tether is moved to Channel 4 for ParB loading. The right panel illustrates a zoomed-in view of the experimental configuration (not to scale).

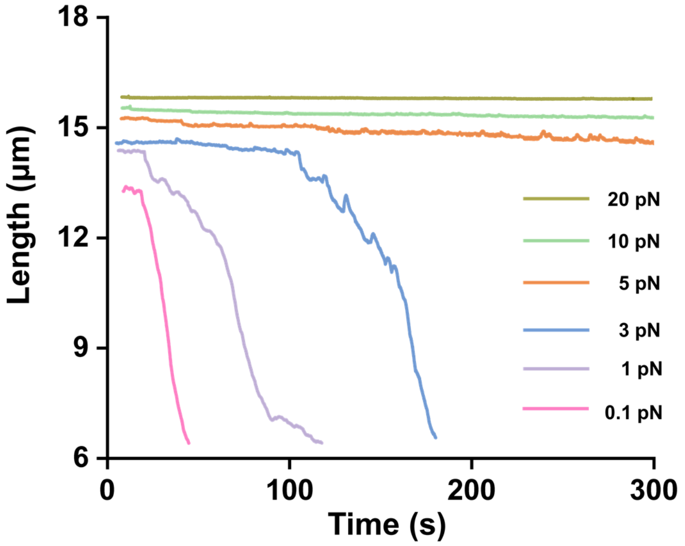

Supplementary Figure S5. ParB-mediated DNA condensation under different forces. Representative DNA length of λ DNA molecule as a function of time in the presence of 250 nM ParB under indicated forces. ParB can condense DNA against a force lower than 3 pN.

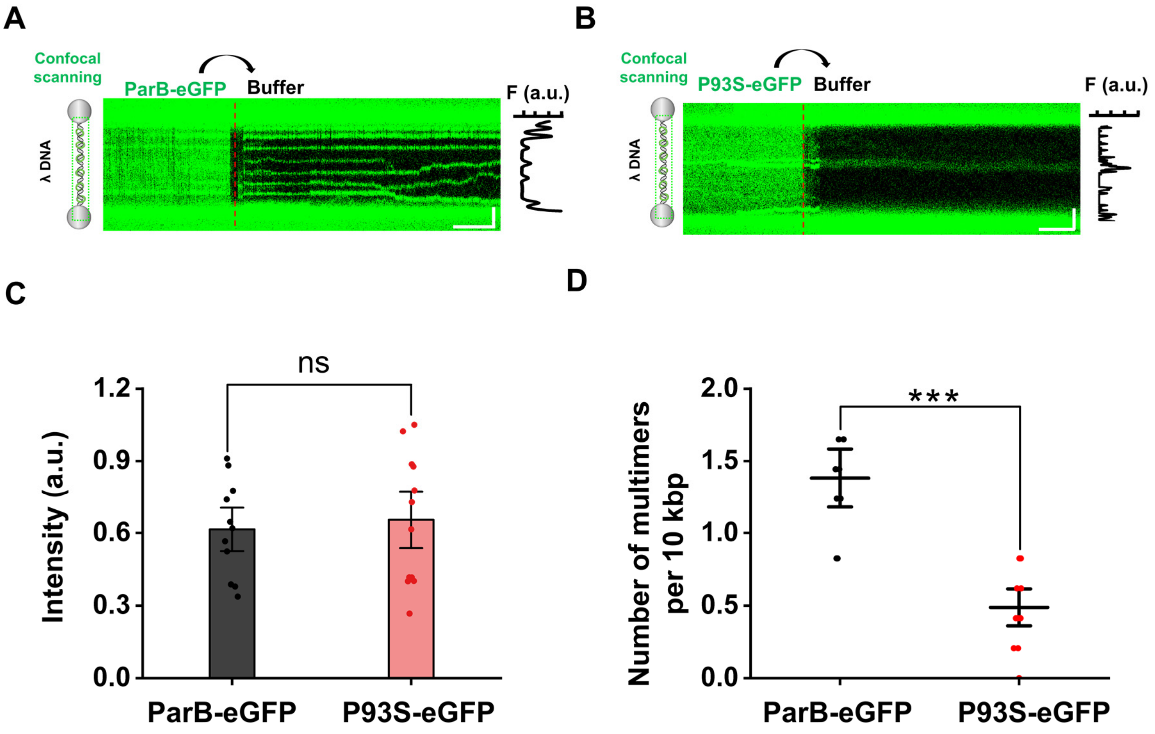

Supplementary Figure S6. DNA binding and multimerization of ParB-eGFP and P93S-eGFP. A and **B.** A representative kymograph of a suspended λ DNA showing 50 nM ParB-eGFP (A) and 50 nM P93S-eGFP (B) binding to the DNA before and after the transportation from the protein channel to the buffer channel. Scale bars, 3 μm and 10 seconds, respectively. The final fluorescent profile of ParB along the DNA is shown alongside the kymograph. **C.** Overall DNA-bound ParB-eGFP and P93S-eGFP intensities in the protein channel. The data are shown as mean ± SEM. n = 11 and 12, respectively. **D.** The average number of ParB multimers formed along the λ DNA per 10 kbp in the buffer channel after a 1-minute incubation in the protein channel. n = 7 and 8, respectively. ***$p < 0.001$.

These findings suggest that whereas P93S maintains the ability to bind DNA, its multimerization ability is attenuated.

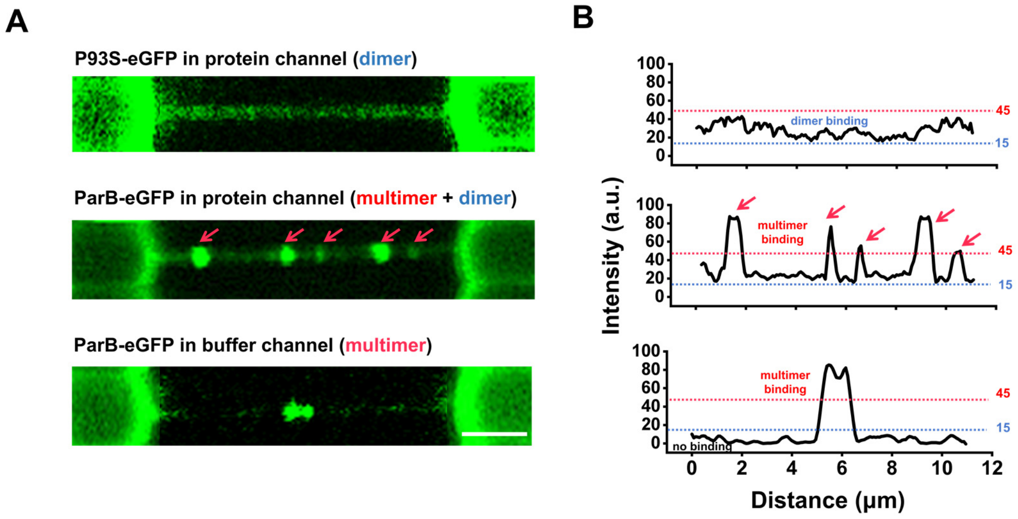

Supplementary Figure S7. Determination of the multimeric status of DNA-bound ParB. A. Representative confocal images of ParB-eGFP or P93S-eGFP in different multimeric forms binding to λ DNA. **B.** The fluorescence profiles of the DNA-bound ParB-eGFP or P93S-eGFP in A. The red arrows highlight the ParB multimer. The red and blue dashed lines represent the thresholds for determining ParB multimer and dimer. Scale bar, 2 μm.

The uniform DNA binding profile of P93S-eGFP allowed us to measure the ParB dimer's fluorescence intensity, typically within 15 and 45 per pixel. On the other hand, the multimeric ParB typically has a fluorescence intensity of over 45 per pixel. Accordingly, we set a threshold of 45 to differentiate between ParB dimers and multimers. The fluorescence intensity below 15 was considered naked DNA without ParB binding.

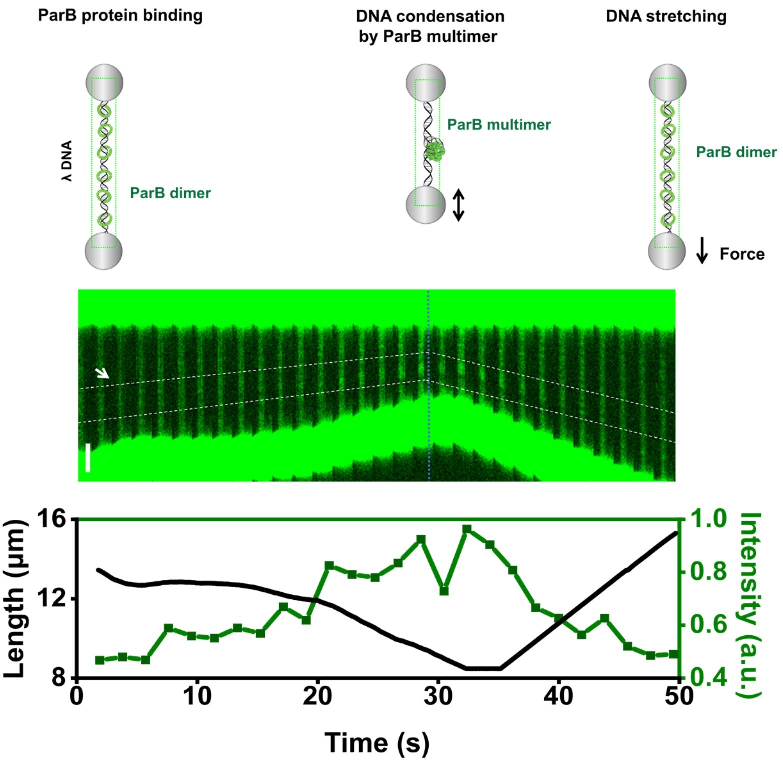

Supplementary Figure S8. Reveral of ParB multimerization by DNA stretching. The cartoons shown above the kymography illustrate the experimental procedures. After the gradually formed ParB condensate started to condense DNA under 0.1 pN, the DNA was stretched by moving the steered trap away from the fixed trap at 0.1 μm/second. The representative kymograph shows the fluorescent signal of ParB-eGFP along the λ DNA during DNA condensation and stretching. The corresponding DNA length (black) and ParB-eGFP multimers (green) intensity are presented below the kymograph. The white arrow indicated the gradually formed ParB multimer. Scale bar, 3 μm. n = 6.

The initially aggregated ParB is correlated with DNA condensation. In addition, based on the analysis of the fluorescence intensity, stretching the condensed DNA reversed ParB multimerization. These findings suggest that DNA condensation is a result of ParB multimerization.

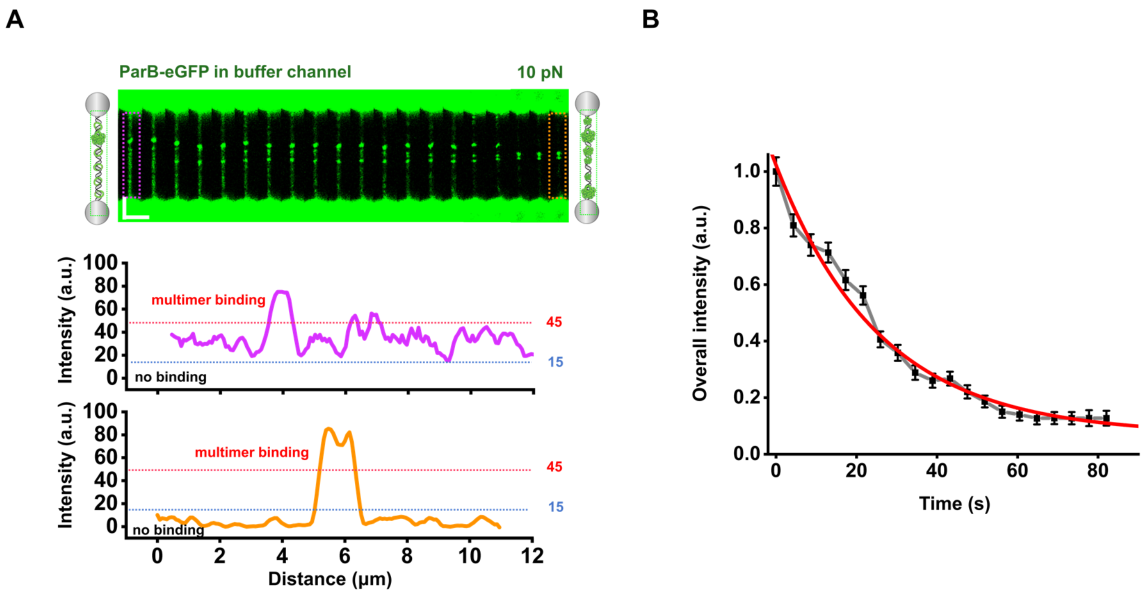

Supplementary Figure S9. Dissociation of ParB dimers and maintenance of ParB multimers on λ DNA under 10 pN. A. A representative kymograph shows the fluorescence signal of DNA-bound ParB-eGFP as a function of time under 10 pN. The fluorescence profiles of the first and last frames are shown below the kymograph. Scale bar, 3 μm and 5 s. **B.** Time-evolution of the overall fluorescence intensity along the DNA.

From the fluorescence analysis, both ParB multimers and dimers were bound to DNA right after the transportation of the ether to the buffer channel. However, after 1 minute and 30 seconds, up to 88% of ParB proteins dissociated, and the remaining DNA-bound ParB proteins were mainly in multimeric forms.

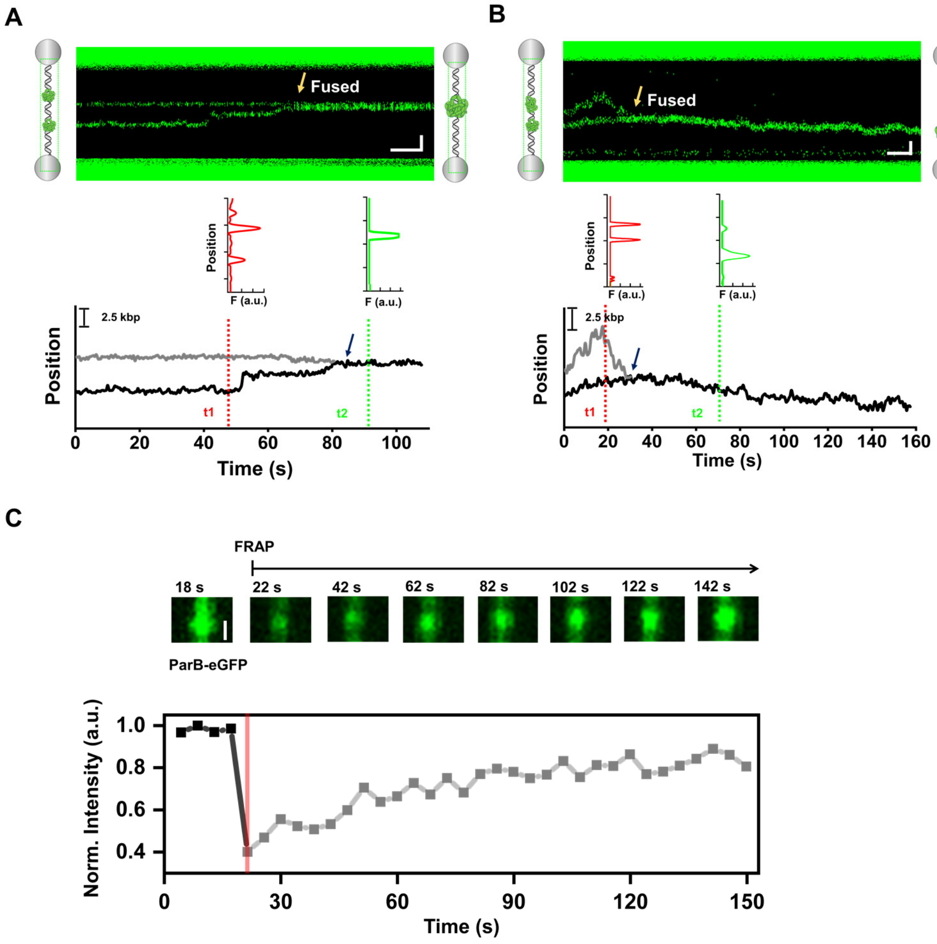

Supplementary Figure S10. ParB multimers have the characteristics of fusion and fluorescence recovery after photobleaching. A. Representative kymographs and real-time trajectories of ParB multimers demonstrate the fusion of two multimers. The fused multimer remained immobile upon the collision. **B.** Representative kymographs and real-time trajectories of ParB multimers illustrate the fusion of two multimers. The fused multimer continued diffusion on DNA after the collision. Scale bars, 5 μm and 10 s, respectively. **C.** Snapshots of the fluorescence images of a single DNA-bound ParB-eGFP multimer at different time points before and after the photobleaching in the protein channel. The quantified fluorescence intensity is shown below. Scale bar, 0.5 μm. Single-molecule photobleaching traces were extracted from raw images via an ImageJ macro. The Origin software was subsequently used to analyze the fluorescence intensity of the obtained images as a function of time.

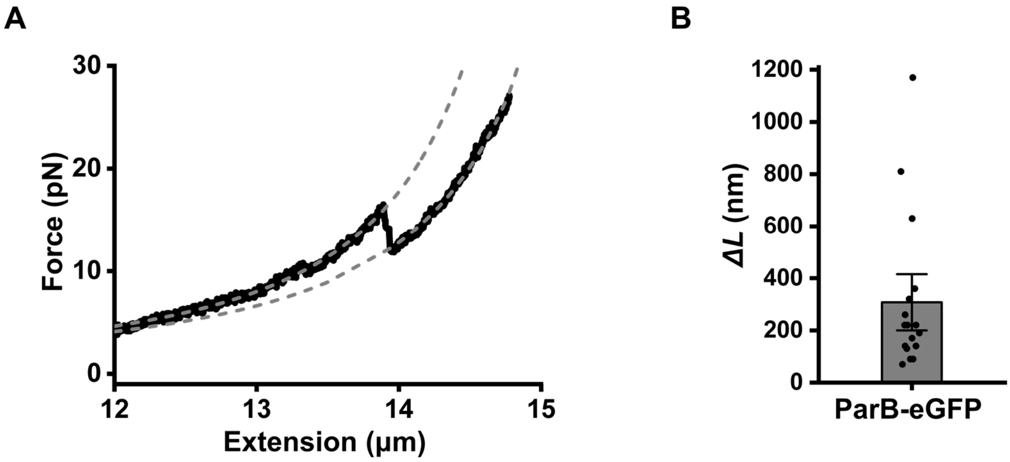

Supplementary Figure S11. Measurements of DNA length change in a sudden force-dropping event. A. A representative force–extension curve of a DNA bound by ParB multimers. The dashed grey lines indicate the theoretical WLC curves before and after the force-dropping events. *ΔL* is calculated as the difference in the contour length of the two fitting curves. **B.** Statistics of *ΔL.* The data are shown as mean ± SEM. n = 17.

Briefly, abrupt, unsmooth changes in the force–extension curve were identified first. By applying the Worm-Like Chain (WLC) model, we measured the contour length of the DNA before and after the force-dropping event in the force-extension curve. This difference in the contour length reflects the size of the looped DNA formed by the ParB multimer.

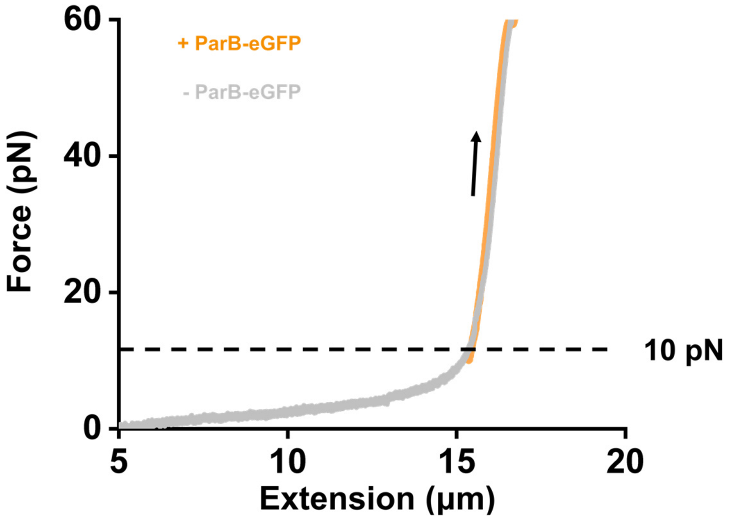

Supplementary Figure S12. The force–extension curve of a ParB-eGFP-coated DNA in the force range of 10 - 60 pN. A representative force–extension curve of a ParB-eGFP-coated (orange) or naked (grey) DNA. A force of 10 pN and above was maintained on the DNA to prevent DNA condensation. In this condition, ParB-eGFP coating on DNA does not alter the mechanical response of the DNA.

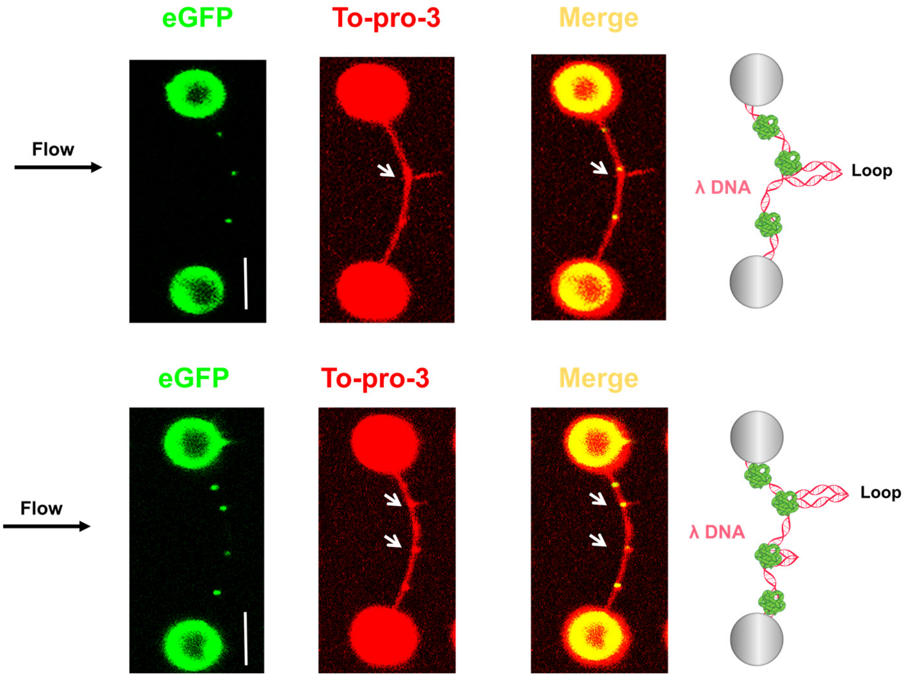

Supplementary Figure S13. Direct observation of DNA loops mediated by ParB-eGFP multimer. Representative images of suspended DNA (red) and DNA-bound ParB-GFP (blue) under a vertical flow. The merged images are shown on the right. Scale bar, 5 μm.

To provide direct evidence for ParB multimer looping DNA, we designed and conducted a single-molecule experiment, where we applied a vertical flow to a suspended DNA tether. This flow allowed the extension of the potentially looped DNA structures for fluorescence imaging. As shown in the two representative confocal images, we observed looped DNA structures with a ParB multimer near the DNA junction. This dataset further corroborates the ability of ParB multimers to loop DNA.

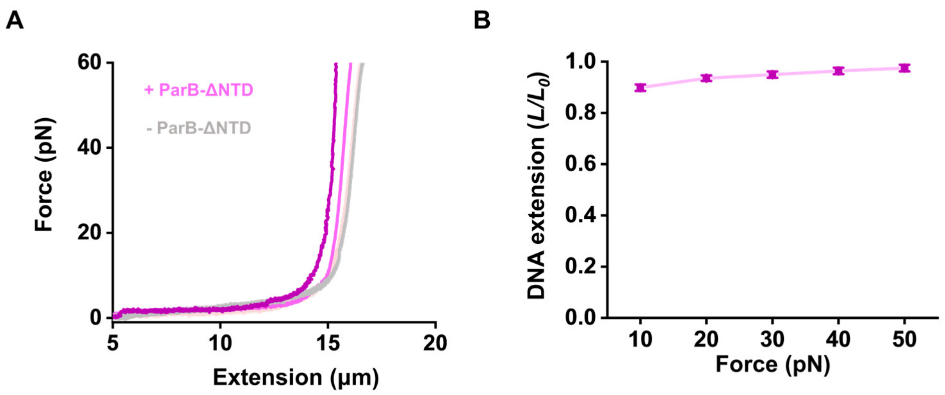

Supplementary Figure S14. DNA condensation in the presence of ParB-ΔNTD-eGFP. **A.** Representative force–extension curves of λ DNA in the presence (pink) and absence (grey) of 250 nM ParB-ΔNTD-eGFP in protein channel (purple lines). **B.** The normalized DNA extension (L/L_0) under different forces in the presence of ParB-ΔNTD-eGFP. L_0 is the original length of naked λ DNA. The data are shown as mean ± SEM. n = 9.

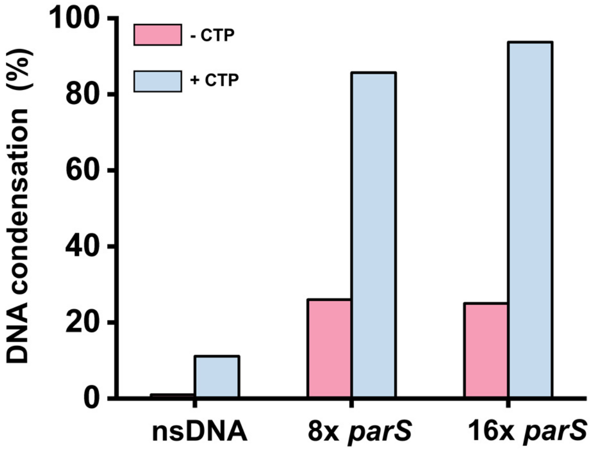

Supplementary Figure S15. *ParS* and CTP facilitate DNA condensation by ParB. The percentage of ns, 8x, and 16x *parS*-containing DNA templates condensed in the presence of 50 nM PaB-eGFP without or with 2 mM CTP (n = 20, 9, 22, 17, 10, and 28, respectively).

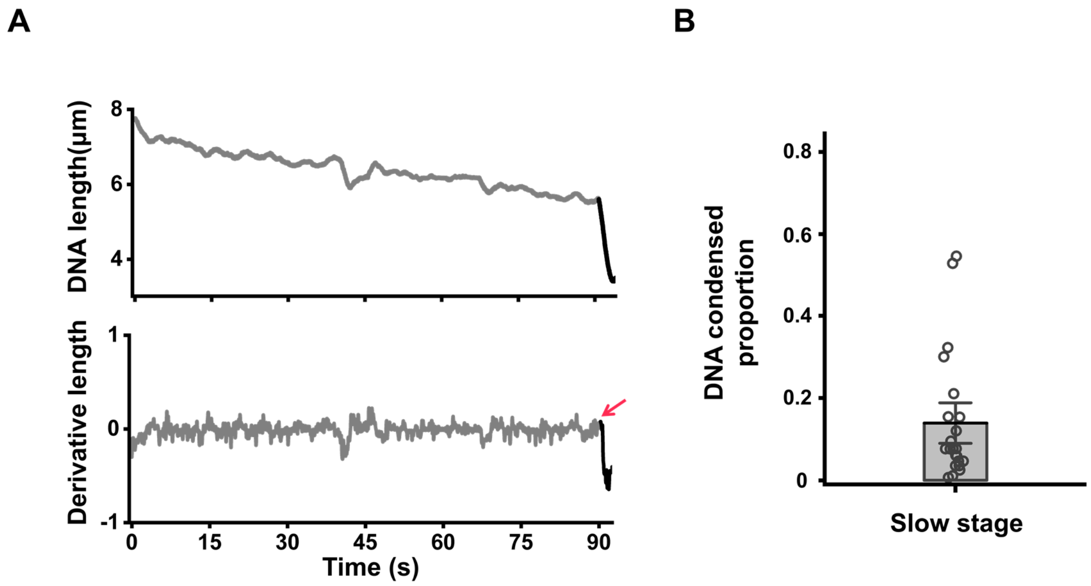

Supplementary Figure S16. Analysis of the two-step DNA condensation process. A. The DNA length and first-order derivative as a function of time during condensation. The red arrow highlights the transition between the slow and fast condensation stages (gray for slow DNA condensation and black for fast DNA condensation). **B.** The DNA compaction ratio in the slow condensation regime. This analysis provides a statistical overview of the degree to which *parS* DNA condenses during the initial stage of the process.

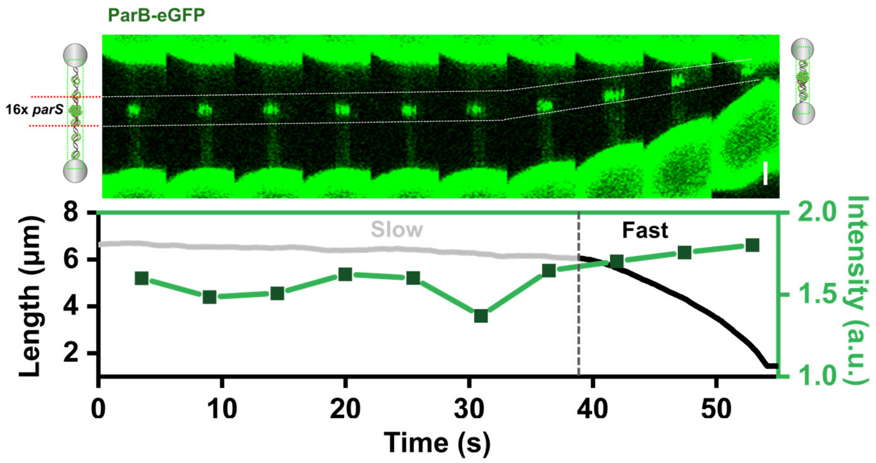

Supplementary Figure S17. The condensation of 16x *parS*-containing DNA in the presence of 50 nM ParB-eGFP and 2 mM CTP. A representative kymograph of the 16x *parS* DNA shows the ParB-eGFP (50 nM) signal under 0.1 pN. The corresponding DNA length (gray for slow DNA condensation and black for fast DNA condensation) and the intensity of the ParB-eGFP multimer (green) are shown below the kymograph. The dotted white line highlights the ParB multimer at the *parS* sites. Scale bar, 2 μm.

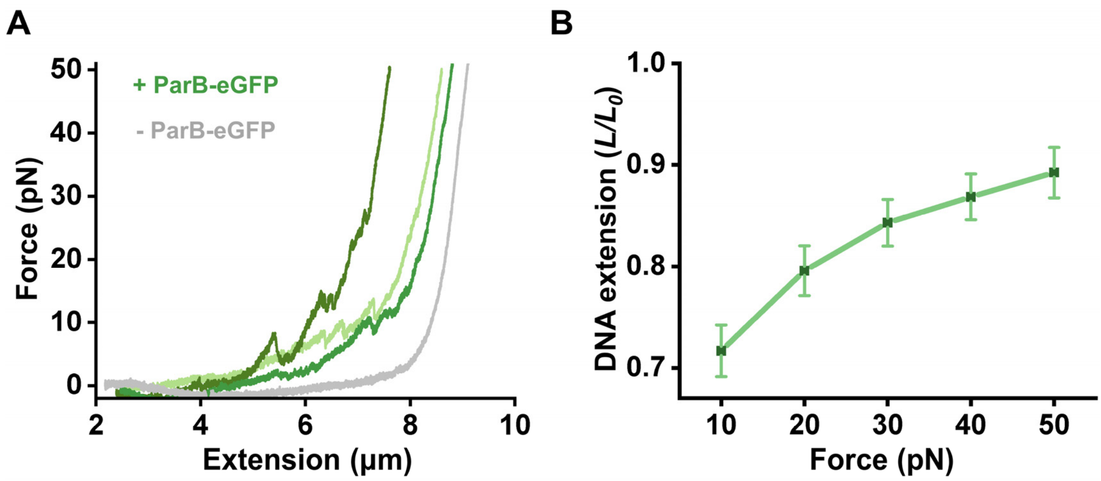

Supplementary Figure S18. ParB multimers at *parS* sites loop distal DNA. A. Representative force–extension curves of the 8x *parS*-containing DNA in the presence (green) and absence (gray) of ParB multimer at *parS*. **B.** The normalized DNA extension (L/L_0) under different forces with ParB multimer binding. L_0 is the original length of naked 8x *parS* containing DNA. The data are shown in mean ± SEM. n = 10. The force-dropping events in this condition indicate that the ParB multimer at *parS* can also loop distant DNA.

Supplementary Table S1. Sequences of oligonucleotides and primers used.

Segment	Oligonucleotide or Primer	Sequence
Cy5-*parS*	Upper	5'- /Cy5/TCGATGCATACGTGACTGTTTCACGTGAAACATCGATGCATACGTGACATTC - 3'
	Lower	5' - GAATGTCACGTATGCATCGATGTTTCACGTGAAACAGTCACGTATGCATCGA - 3'
Cy5-nsDNA	Upper	5' - /Cy5/CGAAGGCTACGTCCAGGAGCGCACCATCTTCTTCAAGGACGACGGCAACTACAAGACC - 3'
	Lower	5' - GGTCTTGTAGTTGCCGTCGTCCTTGAAGAAGATGGTGCGCTCCTGGACGTAGCCTTCG - 3'
L1	Forward (L1)	5' - biotin- AGTGGAAGCTGCTGGACACTGC - 3'
	Reverse (L1)	5' - CCGCTCGAGTCCACAAATACATCCTTGAAGGTCACCAACGC - 3'
L2	Forward (L2)	5' - CCGCTCGAGCAGGACCGGTTCTAGATCAGGCTC - 3'
	Reverse (L2)	5' - biotin- AAACCCGTTGCGAAAAAGAACGTTCACG - 3'
L3	Forward (L3)	5' - CCGCTCGAGTAAGCAGGTCAGTGCGTACGCCAT - 3'
	Reverse (L3)	5' - biotin- TGATAAGCAGAATGGCATCGTTCC - 3'

The *parS* motifs are colored red.
